# The effect of alkali-soluble lignin on purified core cellulase and hemicellulase activities during hydrolysis of extractive ammonia-pretreated lignocellulosic biomass

**DOI:** 10.1098/rsos.171529

**Published:** 2018-06-27

**Authors:** Linchao Zhou, Leonardo da Costa Sousa, Bruce E. Dale, Jia-Xun Feng, Venkatesh Balan

**Affiliations:** 1 State Key Laboratory for Conservation and Utilization of Subtropical Agro-bioresources, College of Life Science and Technology, Guangxi University, Nanning 530004, People's Republic of China; 2 DOE Great Lakes Bioenergy Research Center (GLBRC), Biomass Conversion Research Laboratory (BCRL), Department of Chemical Engineering and Materials Science, Michigan State University, Lansing, MI 48910, USA; 3 Department of Engineering Technology, Biotechnology Division, School of Technology, University of Houston, Houston, TX 77004, USA

**Keywords:** ammonia treatment, lignin inhibition, cellulase, hemicellulase, enzyme synergy

## Abstract

Removing alkali-soluble lignin using extractive ammonia (EA) pretreatment of corn stover (CS) is known to improve biomass conversion efficiency during enzymatic hydrolysis. In this study, we investigated the effect of alkali-soluble lignin on six purified core glycosyl hydrolases and their enzyme synergies, adopting 31 enzyme combinations derived by a five-component simplex centroid model, during EA-CS hydrolysis. Hydrolysis experiment was carried out using EA-CS(−) (approx. 40% lignin removed during EA pretreatment) and EA-CS(+) (where no lignin was extracted). Enzymatic hydrolysis experiments were done at three different enzyme mass loadings (7.5, 15 and 30 mg protein g^−1^ glucan), using a previously developed high-throughput microplate-based protocol, and the sugar yields of glucose and xylose were detected. The optimal enzyme combinations (based on % protein mass loading) of six core glycosyl hydrolases for EA-CS(−) and EA-CS(+) were determined that gave high sugar conversion. The inhibition of lignin on optimal enzyme ratios was studied, by adding fixed amount of alkali-soluble lignin fractions to EA-CS(−), and pure Avicel, beechwood xylan and evaluating their sugar conversion. The optimal enzyme ratios that gave higher sugar conversion for EA-CS(−) were CBH I: 27.2–28.2%, CBH II: 18.2–22.2%, EG I: 29.2–34.3%, EX: 9.0–14.1%, βX: 7.2–10.2%, βG: 1.0–5.0% (at 7.5–30 mg g^−1^ protein mass loading). Endoglucanase was inhibited to a greater extent than other core cellulases and xylanases by lignin during enzyme hydrolysis. We also found that alkali-soluble lignin inhibits cellulase more strongly than hemicellulase during the course of enzyme hydrolysis.

## 1. Introduction

Lignocellulosic biomass obtained from agricultural plant residues (e.g. corn stover (CS), wheat straw, sweet sorghum), dedicated energy crops (e.g. switchgrass, miscanthus, energy cane, short-rotation willow) and woody forest residues are considered as sustainable feedstocks to produce fuels and chemicals in a biorefinery [1,2]. Lignocellulosic biomass comprises two major sugar polymers, namely cellulose (a homopolysaccharide comprised of d-glucose units linked together by β-1,4-glucosidic bonds), with a degree of polymerization of 10 000 or higher, and hemicellulose (different heteropolysaccharides containing different combinations of d-glucose, d-galactose, d-mannose, d-xylose, l-arabinose, d-glucuronic acid and 4-*O*-methyl-d-glucuronic acid), with a degree of polymerization below 200. Complexation of these sugar polymers with lignin makes biomass highly recalcitrant against invading pathogens [3,4]. Processing steps such as thermochemical pretreatment, enzyme hydrolysis and microbial fermentation are required to convert lignocellulosic biomass to fuels and chemicals [5].

Biomass-degrading enzymes (cellulase and hemicellulase) are commercially produced to hydrolyse cellulose and hemicellulose to fermentable sugars [6]. Lignin is the third major component in plant cell wall that inhibits these enzymes during hydrolysis [7]. Lignin is an aromatic polymer comprised of three mono-lignol monomers, methoxylated to various degrees: *p*-coumaryl alcohol, coniferyl alcohol and sinapyl alcohol. The three primary units, namely phenylpropanoids *p*-hydroxyphenyl (H), guaiacyl (G) and syringyl (S), are randomly linked with aryl ether, ester or through carbon bonds and their ratio varies in grasses, hardwoods and soft wood [8]. Pretreatment is a very important process step that is used to increase the accessibility of cellulose and hemicellulose to biomass-degrading enzymes [9,10]. A dilute ammonia pretreatment is currently used by Dupont in its commercial scale plant at Nevada, Iowa. Removing lignin before biological processing may enable further processing to add value to lignin and also improve sugar conversion during downstream processing [11,12]. Pretreatments such as ionic liquid, organosolv and extractive ammonia (EA), selectively remove lignin during the process. The EA pretreatment process could simultaneously extract up to approximately 45% of the lignin from lignocellulosic biomass and convert native crystalline cellulose I (CI) to a highly digestible cellulose III (CIII) allomorph. Near-quantitative retention of the polysaccharides occurs and enzyme hydrolysis is improved [13]. The lignin stream extracted during the EA process was further fractionated into four major fractions and their structural, thermochemical properties and composition were evaluated [14].

Enzyme cost is currently considered the key technical limitation in producing biofuels from lignocellulosic biomass [15–17]. Six core glycosyl hydrolases present in the commercial enzyme cocktail, including four core cellulases (endoglucanase I (EG I, GH family 7B), cellobiohydrolase I (CBH I, GH family 7A), cellobiohydrolase II (CBH II, GH family 6A) and β-glucosidase (βG, GH family 3)) and two core hemicellulases (endoxylanase (EX, GH family 11) and β-xylosidase (βX, GH family 3)), were widely used for cellulose and hemicellulose hydrolysis [18]. Cellobiohydrolases work on cellulosic chains by cleaving off cellobiose units from the chain ends. CBH I acts on reducing ends and CBH II acts on non-reducing ends, while EG randomly hydrolyses internal glycosidic bonds in the cellulose chains [19,20]. βG hydrolyzes cellobiose to glucose specifically [21], EX cleaves the xylan backbone (β-1,4 xylosidic bonds) and βX hydrolyses xylo-oligomers to xylose [22,23]. These enzymes work synergistically with each other to convert complex carbohydrates into fermentable sugars [24,25]. Preserving the enzyme activities during hydrolysis helps maintain enzyme synergies that enable higher sugar conversion [26]. Limited studies have been carried out to isolate lignin from different pretreatment processes and evaluate their effects on cellulase and hemicellulase during hydrolysis [27–32] or by simply washing the biomass with water to evaluate their performance [33]. Investigating the lignin inhibition on mono- and multi-component cellulases and hemicellulases could help to reduce the enzyme mass loading during biomass hydrolysis, thus reducing the enzyme cost in industrial biorefinery. However, to our knowledge little information is available about how alkali-soluble lignin inhibits individual enzymes during hydrolysis and which individual enzymes or classes of enzymes are most vulnerable to activity loss due to alkali-soluble lignin during hydrolysis.

To answer some of the key questions related to alkali-soluble lignin effects on enzyme inhibition, we evaluated the performance of individual purified enzymes and combinations of these enzymes at varying concentrations in the presence and absence of alkali-soluble lignin during hydrolysis of CS. We then compared the performance of core enzymes with commercial enzymes to identify which enzymes are inhibited the most. We also carried out hydrolysis using pure substrates such as Avicel and beechwood xylan by varying the composition of purified enzyme cocktails in the presence and absence of lignin. Experimental results from these studies have helped us understand which classes of enzymes lose activity most rapidly during hydrolysis when alkali-soluble lignin is present in the substrate. Losing some core cellulase activities in the presence of alkali-soluble lignin is one possible reason for why enzyme synergy is lost during hydrolysis resulting in less sugar production. To our knowledge this study is the first of its kind that evaluated individual enzyme activity and the synergy operating between them in the presence and absence of alkali-soluble lignin during hydrolysis. Fundamental understanding of enzyme activity in the presence of lignin will help to develop more stable enzymes to improve the efficiency of sugar conversion and reduce the cost of producing biofuels.

## 2. Experimental

### 2.1. Substrates and reagents

CS used in this work was pre-milled by passing through 10 mm sieve and was provided by the Great Lakes Bioenergy Research Center (GLBRC). The corn was planted and harvested at the Arlington Research Station (WI, USA) in 2014 and CS had a moisture content of 6.7% (g water/g dry biomass). Carboxymethyl cellulose (CMC, Lot #419273), cellobiose (Lot #C7252), beechwood xylan (Lot #9559) and Avicel (Lot #11365) were all purchased from Sigma (Sigma-Aldrich, St Louis, MO). The *para*-nitrophenyl (*pNP*) based chromogenic substrates used were 4-nitrophenyl-β-d-cellobioside (*pNP*C Lot #N5759), 4-nitrophenyl-β-d-glucopyranoside (*pNP*G Lot #N7006), 4-nitrophenyl-β-d-xylopyranoside (*pNP*X Lot #N2132) and 4-nitrophenyl-α-l-arabinofuranoside (*pNP*Af Lot #N3641). All the above substrates were purchased from Sigma (Sigma-Aldrich).

Pre-cast Nu-PAGE® Novex 4–12% Bis-Tris gels were purchased from Invitrogen (Lot #NP0321BOX). Then the gel was stained with GelCode Blue Stain Reagent purchased from Thermo Fisher Scientific (Lot #KD131759, Rockford, IL, USA). Bicinchoninic acid (BCA) was purchased from Pierce Biotechnology, Rockford, IL, bovine serum albumin (BSA) was purchased from Thermo Fisher Scientific and 4-nitrophenol (*pNP*, Lot #1048) was purchased from Sigma-Aldrich. All buffer salts, mineral acids and routinely used laboratory chemicals were purchased from Fisher Scientific. Anhydrous liquid ammonia used in this work was purchased from Airgas, Michigan.

### 2.2. Preparation of extractive ammonia-treated corn stover

**Figure 1.**
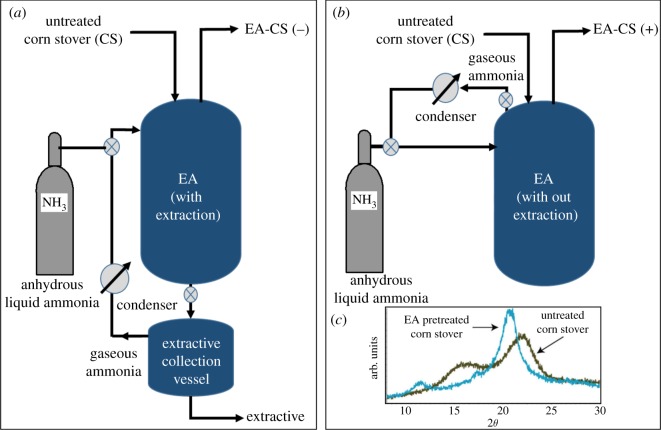
Process flow diagram showing how biomass was pretreated and crystal structure changes that take place in cellulose. (*a*) EA-treated CS after lignin extraction [EA-CS(−)]; (*b*) EA-treated CS without lignin extraction [EA-CS(+)]; (*c*) transformation of crystal structure of cellulose before and after pretreatment.

CS was subjected to EA pretreatment using the following conditions: 6 : 1 ammonia to biomass weight ratio (NH_3_: BM), at 10% (w/w) moisture (dry weight basis) and 120°C for 30 min residence time [13]. Process flow diagram (figure 1) shows how biomass EA-CS(−) and EA-CS(+) were pretreated, and the crystal structure changes that take place in cellulose (CI to CIII). EA-CS(−) was prepared by collecting the soluble extractives in a separate vessel from the bottom of the reactor after completing the pretreatment process and then venting the ammonia from the collection vessel. EA-CS(+) was prepared in the same way as EA-CS(−), except that the ammonia in the reactor was vented from the top of the reactor and thus no lignin extraction occurred. EA-CS(−) had approximately 40% lignin removed (most of the alkali-soluble lignins are removed during EA pretreatment) compared to untreated CS, while the EA-CS(+) had approximately the same lignin content as untreated CS. After completing the pretreatment process, the samples were transferred from the reactor to an aluminium tray and dried in a hood overnight (24 h) to remove residual ammonia bound to biomass. Both pretreated biomass materials were then milled to a particle size less than 100 µm, using a centrifugal mill (model ZM 200, Retsch, Newtown, PA) reported previously [34].

EA pretreatment was performed on CS at low moisture levels, typically around 10% (total weight basis), in three stages: (1) pretreatment, (2) extraction and (3) product/solvent recovery. Stage 1 (pretreatment) is performed in the reactor vessel, in which liquid ammonia is contacted with biomass at a sufficiently high loading (6 : 1 ammonia to biomass loading) to fully immerse the biomass at a defined temperature and residence time. As temperature increases, ammonia pressure builds up until a new vapour–liquid equilibrium is established. It is important to control the reactor volume so that most of the ammonia is in the liquid phase, submerging the biomass, at equilibrium. During this stage, the cellulose–ammonia complex is formed, ester bonds are cleaved and lignin is partly solubilized in the liquid ammonia phase. In Stage 2, EA-pretreated biomass is filtered to separate the ammonia-soluble components from the residual solids. During this stage, lignin is extracted, and CIII is formed from the cellulose–ammonia complex as ammonia is continuously removed from the biomass into an extract-collection vessel. During Stage 3, ammonia is evaporated from the extractives by subjecting the vessel to heat, and the extract is recovered as a dark brown viscous liquid (figure 1).

The extract contains approximately 40% of the total lignin from the untreated CS. This lignin stream is further fractionated by sequential precipitation using ethanol and water at room temperature [14].

### 2.3. Composition analysis

Sugar polymers (glucan, xylan and arabinan), and the lignin content for three CS materials, namely two EA treated CS materials (EA-CS(−) and EA-CS(+)) and the untreated CS, were analysed using the National Renewable Energy Laboratory (NREL) protocol [35]. The compositions are given in table 1.

**Table 1.** Compositions of untreated and two different pretreated CS. Data are average ±s.d. for all experiments in triplicate.

biomass	glucan %	xylan %	arabinan %	lignin %	ash %
untreated CS	37.1 ± 0.3	28.1 ± 0.1	4.2 ± 0.1	12.4 ± 0.5	0.3 ± 0.1
EA-CS(−)	41.9 ± 0.2	30.9 ± 0.1	4.5 ± 0.1	8.3 ± 0.1	0.6 ± 0.1
EA-CS(+)	37.3 ± 0.3	27.4 ± 0.1	4.2 ± 0.1	13.1 ± 0.7	0.2 ± 0.1

### 2.4. Enzyme sources

CBH I, CBH II and EG I were purified from Spezyme CP (Daniso US Inc., Dupont, Rochester, NY). While EX was isolated from Multifect Xylanase (Daniso US Inc.). Both Spezyme CP and Multifect Xylanase were produced using *Trichoderma reesei*. The recombinant β-xylosidase (βX) was expressed in *Pichia pastoris* strain (FGSC #10077) containing the βX gene which was obtained from the Fungal Genetics Stock Center (FGSC) at the University of Missouri (Kansas City, MO). The gene encoding βX was obtained from *Aspergillus nidulans* and integrated into the genome of *P. pastoris X-33* [36]. The commercial β-glucosidase (βG) was from *Aspergillus niger* (Megazyme International Ireland Ltd, Bray, Ireland, EC # 3.2.1.21).

Cellic® CTec2 (192 mg protein ml^−1^) and Cellic® HTec2 (173 mg protein ml^−1^) were generously provided by Novozymes (Franklinton, NC, USA). Cellic® CTec2 was a complex blend of cellulase, β-glucosidase and some hemicellulase; Htec2 was an endoxylanase with cellulase background.

### 2.5. Estimation of protein concentrations

Concentrations of the commercial enzymes Cellic CTec2 and HTec2 were determined by estimating the protein (and subtracting the nonprotein nitrogen contribution) using the Kjeldahl nitrogen analysis method (Ithaca, NY, USA). Purified enzyme concentrations were determined by the BCA method using BSA as standard [37]. Protein samples were first denatured in boiling water for 10 min, electrophoresis was performed using a Novex® XCell SureLock™ Mini-Cell system (Invitrogen, Carlsbad, CA, USA) using pre-cast Nu-PAGE® Novex 4–12% Bis-Tris gels (Invitrogen), the gel was stained with GelCode Blue Strain Reagent (Thermo Fisher Scientific) and de-stained by distilled water for 48 h at room temperature, until specific protein bands appeared.

### 2.6. Enzyme activity assay

The enzyme activity assays were based on a high-throughput microplate-based method as described in previous work [34]. A 2.2 ml deep-well microplate (Lot #780271, Greiner, Monroe, NC) was used to add 250 µl of 1% (w/v) stock substrate (CMC, Avicel, beechwood xylan, cellobiose and xylobiose), 50 µl of 0.5 M citrate buffer (pH 5.0) and 200 µl of appropriately diluted enzyme samples (20 ng to 100 µg well^−1^). The microplates were incubated at 50°C while rotating at 10 r.p.m. for 10 min (for cellobiose), 60 min (for CMC and xylan) or 300 min (for Avicel). The glucose released was estimated using high-performance liquid chromatography (HPLC). One unit of cellobiase was defined as 1 mM glucose released per milligram enzyme per minute under the assay conditions. For CMC, Avicel and xylan-based substrates reducing sugars were estimated using a 3,5-dinitrosalicylic acid (DNS)-based assay [38]. The hydrolysate supernatants were filtered through a 0.45 µm microplate filter (Lot #R6PN00144, Millipore, Ireland) and 50 µl of the supernatant was incubated with 100 µl of DNS reagent in polypropylene microplate wells (Lot #651201, Greiner, NC) at 100°C for 30 min. After the plates were cooled to room temperature, 100 µl of the solution was transferred to a clear, flat-bottom microplate (Lot #353072, Becton Dickinson Labware, NJ, USA) to measure absorbance at 540 nm using a Victor microplate reader (PerkinElmer, MA, USA). Suitable reducing sugar standards (either glucose or xylose standards in the concentration range 0.1–2 g l^−1^) were included for the DNS assay.

One unit of CMCase, Avicelase and xylanase activity was defined as 1 µM reducing sugars (as glucose equivalents for Avicel/CMC and xylose equivalents for xylan) released per milligram enzyme per minute under the respective assay conditions. The assay mixtures containing 80 µl of 1 mM *pNP* substrate, 10 µl of 0.5 M citrate buffer (pH 5.0) and 10 µl of diluted enzymes (20 ng to16 µg per well) in 500 µl microplates were incubated at 50°C with rotation at 10 r.p.m. After 15 min reaction time, 200 µl of 1 M Na_2_CO_3_ was added to assay mixtures to stop the hydrolytic reaction. The amount of *pNP* released was quantified by measuring absorbance (at 420 nm) of *pNP* based standard curve (0.1 to 1 mM). One unit of enzyme activity was defined as 1 mM *pNP* released per milligram enzyme per minute under the assay conditions.

### 2.7. Lignin inhibition on different enzyme combinations

An enzyme hydrolysis model for EA-CS(−) and EA-CS(+) was designed as reported previously [24]. Here, the model employed in this study was to evaluate the effect of alkali-soluble lignin on various enzyme combinations; it was also aimed at finding the optimal core enzyme ratios at highest glucose yield for EA-CS(−). A five-component simplex centroid enzyme mixture design, including CBH I, CBH II, EG I, EX and βX, was firstly generated using Minitab (v. 17.0, Minitab Inc., State College, PA) statistical software. Thirty-one enzyme combinations of five core enzymes were determined at three different total protein loadings (7.5, 15 and 30 mg g^−1^ glucan), with βG loaded separately at 10% supplementation of other five enzymes to convert glucose oligomers released by other enzymes into glucose, thereby preventing the inhibition of glucose oligomers.

The hydrolysis experiments were performed in 1 ml deep-well microplates at 0.2% (w/w) total glucan loading for both EA-CS(−) and EA-CS(+), along with 50 mM citrate buffer (pH 5.0) with total reaction volume of 500 µl [34]. The microplates were incubated at 50°C in rotating oven at 10 r.p.m. for 24 h; all assays were in triplicate and were performed as mixtures at fixed protein mass loadings per gram of glucan in the CS. After enzymatic hydrolysis, enzymes were de-activated at 100°C for 10 min, the samples were then filtered and the glucose and xylose released during hydrolysis were assayed by a HPLC system, equipped with a Shimadzu refractive index detector. The hydrolysate and monomeric sugar samples were analysed using Bio-Rad Aminex HPX-87H column using 5 mM sulfuric acid as mobile phase with a flow rate of 0.6 ml min^−1^ following the appropriate NREL protocols [35–37,39]. The injection volume was 10 µl with a run time of 12 min. Mixed sugar standards were used to quantify cellobiose and other monosaccharides (glucose, xylose, galactose, arabinose and mannose). All the HPLC data for glucose and xylose yields were further analysed to generate a mixture regression statistical model to predict optimum enzyme mixtures.

### 2.8. Enzyme optimization for extractive ammonia corn stover hydrolysis

Results of glucose yields for 31 enzyme combinations were further analysed by Minitab to predict the optimal enzyme ratios of five core enzymes, namely CBH I, CBH II, EG I, EX and βX, for both EA-CS(−) and EA-CS(+), at three different enzyme mass loadings (with βG loaded at 10% supplementation). Meanwhile, the highest sugar yields (glucose/xylose) for both EA-CS(−) and EA-CS(+) were also predicted by Minitab. The βG ratio was then optimized after the optimization of the other five enzymes (varying the βG ratios with other five enzymes loaded at fixed optimal ratios).

### 2.9. Lignin inhibition studies using optimal enzymes

The lignin inhibition on optimal enzymes experiments were performed in 4 ml reaction volume cylindrical glass bottles, with the following hydrolysis conditions: 50°C, citrate buffer at pH 5.0, 200 r.p.m. using a shaking incubator, and 24 h. With 1% (w/w) Avicel and beechwood xylan mass loading, fixed glucan loading (0.4%) for both EA-CS(−) and EA-CS(+). The alkali lignin F3 (WIL, water-insoluble and ethanol-soluble), prepared using a previously reported protocol [14], was added to pure Avicel and beechwood xylan at fixed 1 : 5 ratio (w/w), and also at fixed 1 : 5 ratio (lignin/biomass mass) for EA-CS(−). The lignin-rich product stream (F3) was practically free of carbohydrates, and characterized by its good yield, low molecular weight, ethanol solubility, high (92%) lignin content and high proportion of intact, native lignin functionality (e.g. β-*O*-4 linkages). All six purified core enzyme were using the optimal enzyme ratios for EA-CS(−) at 15 mg g^−1^ (with βG fixed at 5% of other five total enzyme mass). The total enzyme mass loading was 15 mg g^−1^ glucan for EA-CS(−) and EA-CS(+), and 15 mg g^−1^ substrate for Avicel and beechwood xylan, respectively.

### 2.10. Modelling study

The effects of lignin on six purified core enzymes during EA-CS hydrolysis were investigated in 31 enzyme combinations and at three total protein loadings by using a five-component simplex centroid mixture design model [24]. Minitab (v. 17.0, Minitab Inc.) statistical software was used to create a suitable mixture optimization design and analyse responses. In a mixture problem with *q* factors, it is common to define proportion variables *x_i_*, for *i *= 1, *2*, … ,*q*, where *x_i_* ≥ 0 represents the proportion of ingredient *i* in the mixture and *x*_1_ + *x*_2_ +* … *+* x*_*i*_ +* … *+ *x_q_* = 1. The proportion variable allows one to consider a particular mixture experiment as a geometric point. In particular, the set of all points (*x*_1_, *x*_2_* *… ,*x_q_*) whose coordinates satisfy *x_i_* ≥ 0 and *x*_1_* *+ *x*_2_ +* *…* *+ *x*_*i*_ +* *…* *+ *x_q_* = 1 is called a *q*-dimensional simplex.

In this work, a five-component simplex centroid mixture design was generated for CBH I, CBH II, EG I, EX and βX, withβG loaded separately at 10% supplementary for all 31 enzyme combinations at three total enzyme loadings (7.5, 15 and 30 mg g^−1^ glucan), because it could convert oligomeric sugars (mainly cellobiose) released by other enzymes into glucose. With sugar yields determined, the glucan and xylan conversions were calculated to reflect the effects of alkali-soluble lignin on mono- and multi-component enzymes during EA-CS hydrolysis. Furthermore, these data were then analysed by the Minitab software to generate a mixture regression statistical model and used to predict optimum mixture compositions that maximize glucan and xylan digestibility for EA-CS(−) and EA-CS(+). The βG ratio was then re-optimized after the optimization of the other five enzymes. Hydrolysis experiments were carried out in triplicate, with specific parameters of average value, standard deviation, *p*-value, *R*^2^ and *T*-test(*p*), which are listed in the text.

## 3. Results and discussion

### 3.1. Enzyme purity and activities

The six purified core enzymes employed in this work showed single protein bands based on the SDS–PAGE result (electronic supplementary material, figure S1). The purities of all six enzymes are greater than 99% based on the quantification of the SDS–PAGE gel band intensity using UN-SCAN-IT gel™ software.

The purified enzymes were evaluated for their hydrolytic activity on various substrates. The activities of all enzymes were evaluated on processed polymeric substrates (i.e. Avicel, beechwood xylan, CMC) and synthetic substrate (*pNP-*based chromogenic bound sugars). The results of these activity assays for all purified enzymes are shown in electronic supplementary material, table S1. The endo-enzyme EG I had high specific activity on CMC (1.74 U) and xylan (5.01 U); comparable to EX activity on xylan (8.69 U). Interestingly, endoxylanase (and xylo-oligomerase) activity for EG I was reported previously [40], in addition to activity on Avicel (0.09 U), suggesting that this enzyme plays a dual role in hydrolysing glucan and xylan in pretreated biomass. We also found that EG I showed activity on a *pNP*C-based chromogenic substrate. Among the exo-acting enzymes, CBH I and CBH II had significant activity on Avicel (0.13 and 0.19 U, respectively). This is not surprising, considering Avicel has a significant proportion of amorphous cellulose (nearly 20–30%) [41].

Purified CBH I showed much lower *pNP*C activity (0.00066 U) than did EG I, while CBH II had no detectable activity on *pNP*C. Enzymes βG and βX did not show appreciable activity on any of the polysaccharide-based substrates, but showed significant activity on cellobiose (221.45 U) and xylobiose (54.59 U), respectively. In addition to this, βG and βX also showed significant activity on *pNP*G (8.58 U) and *pNP*X (1.27 U), respectively. The enzyme βG also had significant activity on *pNP*C (1.30 U). Similarly, βX has trace activity on *pNP*G (0.0071 U), but no detectable activity on cellobiose. βX activity on *pNP*Af (0.15 U) would indicate α-arabinofuranosidase cross-activity. Similar cross-activity has been reported earlier for certain GH 3 β-xylosidases [42]. Other enzymes such as CBH II and EX had no detectable activities on any of the chromogenic *pNP* substrates. These enzyme activity assay results show that the purified enzymes are active on their respective substrates as reported in prior research [24].

### 3.2. Lignin inhibition results on various core enzyme combinations

Thirty-one enzyme combinations including CBH I, CBH II, EG, EX and βX were tested for hydrolysis of EA-CS(−) and EA-CS(+), respectively, with the enzyme βG loaded separately at additional 10% of other five enzymes mass loading to prevent cellobiose inhibition. Enzyme combinations were designed by Minitab, and were tested at three fixed protein mass loadings (7.5, 15 and 30 mg g^−1^ glucan). Enzyme hydrolysis results of glucose and xylose yields in triplicate for both EA-CS(−) and EA-CS(+) are given in tables 2 and 3. The standard deviations associated with the experiments were mostly less than 2% among triplicates.

**Table 2.** Glucan conversion after 24 h hydrolysis for two different pretreated CS samples at three different protein loadings, while keeping βG loading at 10% supplementation in all experiments. Average (avg.) and standard deviation (s.d.) were based on experiments that were done in triplicate.

						glucan conversion for EA-CS(−)						glucan conversion for EA-CS(+)					
	purified enzymes					7.5 mg g^−1^		15 mg g^−1^		30 mg g^−1^		7.5 mg g^−1^		15 mg g^−1^		30 mg g^−1^	
no.	CBH I	CBH II	EG I	EX	bX	avg. (%)	s.d. (%)	avg. (%)	s.d. (%)	avg. (%)	s.d. (%)	avg. (%)	s.d. (%)	avg. (%)	s.d. (%)	avg. (%)	s.d. (%)
1	1	0	0	0	0	5.6	0.1	9.7	0.3	18.9	0.4	5.0	0.1	8.9	0.0	18.3	0.1
2	0	1	0	0	0	5.8	0.1	7.5	0.1	10.0	0.2	5.1	0.2	6.2	0.1	8.2	0.0
3	0	0	1	0	0	16.8	0.2	20.8	0.3	25.8	0.3	13.4	0.2	16.5	0.1	21.7	0.0
4	0	0	0	1	0	4.5	0.2	6.0	0.2	7.6	0.1	4.6	0.1	5.7	0.3	7.0	0.2
5	0	0	0	0	1	1.7	0.2	2.2	0.3	2.5	0.1	2.2	0.1	2.6	0.0	3.0	0.1
6	0.5	0.5	0	0	0	8.4	0.2	14.1	0.2	24.6	0.2	7.1	0.1	12.1	0.1	22.5	0.2
7	0.5	0	0.5	0	0	37.2	0.2	54.6	0.0	73.9	0.2	35.8	0.4	53.2	0.5	69.1	2.5
8	0.5	0	0	1	0	11.5	0.2	22.0	0.4	40.5	0.3	10.9	0.0	22.1	0.2	41.0	0.2
9	0.5	0	0	0	1	3.7	0.1	6.2	0.2	11.9	0.1	3.8	0.2	5.7	0.1	11.2	0.2
10	0	0.5	0.5	0	0	31.0	0.4	42.9	0.2	58.7	0.4	27.7	0.5	39.0	0.7	56.7	0.8
11	0	0.5	0	1	0	13.3	0.1	19.9	0.2	29.8	0.3	11.9	0.3	17.7	0.0	28.9	0.3
12	0	0.5	0	0	1	4.7	0.2	6.3	0.1	8.2	0.2	4.6	0.3	5.4	0.2	7.0	0.1
13	0	0	0.5	1	0	16.2	0.1	19.3	0.0	23.4	0.2	12.8	0.4	15.4	0.7	19.5	0.2
14	0	0	0.5	0	1	14.6	0.2	17.9	0.1	22.2	0.1	11.5	0.3	14.0	0.2	17.7	0.1
15	0	0	0	1	1	3.4	0.1	5.1	0.2	6.5	0.0	3.8	0.2	4.9	0.0	6.3	0.2
16	0.33	0.33	0.33	0	0	52.3	0.3	69.4	0.3	82.4	1.7	50.6	0.2	65.8	4.0	77.8	4.8
17	0.33	0.33	0	0	0	26.1	0.1	45.9	0.2	71.4	0.9	25.0	0.4	45.7	0.8	64.0	0.4
18	0.33	0.33	0	0	0	7.0	0.2	11.5	0.1	20.0	0.2	5.9	0.0	9.5	0.2	17.8	0.2
19	0.33	0	0.33	0	0	41.5	0.3	58.9	0.1	79.4	1.4	38.9	0.2	56.4	1.4	71.1	3.4
20	0.33	0	0.33	0	0	33.6	0.1	49.7	0.5	71.2	1.7	31.7	0.3	48.0	0.5	63.4	1.8
21	0.33	0	0	0	0	8.8	0.2	16.3	0.2	30.7	0.0	8.7	0.1	16.1	0.1	30.7	0.5
22	0	0.33	0.33	0	0	31.9	0.1	43.6	0.2	58.8	0.5	29.2	0.2	40.0	0.5	56.1	1.3
23	0	0.33	0.33	0	0	27.1	0.2	37.7	0.1	53.0	0.5	23.7	0.5	33.9	0.4	49.3	0.5
24	0	0.33	0	0	0	11.0	0.2	16.5	0.3	24.7	0.2	9.5	0.2	15.1	0.0	23.3	0.3
25	0	0	0.33	0	0	14.2	0.4	17.3	0.3	21.0	0.2	11.2	0.5	13.2	0.3	16.6	0.4
26	0.25	0.25	0.25	0	0	60.8	0.3	78.0	0.4	93.9	1.5	58.8	0.4	74.4	2.2	80.4	2.5
27	0.25	0.25	0.25	0	0	49.8	1.2	68.7	0.8	84.9	0.8	47.0	1.3	63.2	0.5	73.9	1.8
28	0.25	0.25	0	0	0	20.9	0.2	38.4	0.3	63.7	1.1	20.3	0.5	38.4	0.1	61.5	0.8
29	0.25	0	0.25	0	0	36.5	0.3	53.0	0.1	73.8	0.6	35.3	1.4	52.0	0.0	72.5	1.3
30	0	0.25	0.25	0	0	28.2	0.3	39.0	0.4	53.9	0.6	25.6	0.4	36.2	0.9	51.9	1.4
31	0.2	0.2	0.2	0	0	55.0	0.5	75.7	0.5	89.8	1.4	53.9	0.7	74.1	1.9	89.5	3.9
32	Ctec2/Htec2 7/3					73.5	0.1	88.5	0.7	99.5	1.4	65.5	1.3	81.0	0.7	94.1	1.9

**Table 3.** Xylan conversion after 24 h hydrolysis for two different pretreated CS samples at three different protein loadings, while keeping βG loading at 10% supplementation in all experiments. Average (avg.) and standard deviations (s.d.) were based on experiments that were done in triplicate.

						xylan conversion for EA-CS(−)						xylan conversion for EA-CS(+)					
	purified enzymes					7.5 mg g^−1^		15 mg g^−1^		30 mg g^−1^		7.5 mg g^−1^		15 mg g^−1^		30 mg g^−1^	
no.	CBH I	CBH II	EG I	EX	bX	avg. (%)	s.d. (%)	avg. (%)	s.d. (%)	avg. (%)	s.d. (%)	avg. (%)	s.d. (%)	avg. (%)	s.d. (%)	avg. (%)	s.d. (%)
1	1	0	0	0	0	2.3	0.1	3.6	0.1	5.8	0.3	2.1	0.0	3.3	0.0	5.3	0.1
2	0	1	0	0	0	1.3	0.1	1.5	0.2	2.1	0.2	1.5	0.2	1.7	0.1	2.2	0.2
3	0	0	1	0	0	6.9	0.5	10.7	0.2	14.2	0.4	6.6	0.2	9.8	0.1	13.1	0.1
4	0	0	0	1	0	13.4	0.7	19.3	0.7	24.2	0.6	12.5	0.4	17.2	1.1	22.5	1.5
5	0	0	0	0	1	2.4	0.2	2.8	0.4	3.3	0.1	2.4	0.1	2.9	0.2	3.2	0.1
6	0.5	0.5	0	0	0	1.8	0.2	2.9	0.1	4.3	0.3	2.0	0.1	2.9	0.1	4.4	0.2
7	0.5	0	0.5	0	0	5.5	0.1	8.3	0.2	11.0	0.3	5.1	0.2	7.6	0.3	10.3	0.2
8	0.5	0	0	1	0	10.6	0.4	14.6	0.2	18.1	0.5	8.9	0.2	13.3	0.2	16.2	0.4
9	0.5	0	0	0	1	5.5	0.2	8.3	0.1	12.3	0.1	5.1	0.2	7.3	0.1	11.1	0.2
10	0	0.5	0.5	0	0	5.6	0.2	8.3	0.1	11.3	0.2	5.2	0.2	7.2	0.3	10.5	0.3
11	0	0.5	0	1	0	10.7	0.3	15.0	0.1	18.3	0.2	9.7	0.5	12.8	0.6	16.8	0.7
12	0	0.5	0	0	1	2.3	0.1	3.3	0.2	4.1	0.1	2.6	0.2	3.3	0.5	4.0	0.1
13	0	0	0.5	1	0	11.1	0.1	15.4	0.2	19.3	0.4	9.8	0.8	14.1	1.6	19.1	0.2
14	0	0	0.5	0	1	29.3	0.2	33.7	0.3	39.5	0.3	24.7	0.6	29.7	0.5	34.7	0.6
15	0	0	0	1	1	40.3	0.7	45.0	0.9	48.6	0.7	36.6	3.4	40.9	0.5	45.0	2.6
16	0.33	0.33	0.33	0	0	4.8	0.1	7.2	0.1	10.1	0.1	4.3	0.1	6.3	0.2	9.3	0.1
17	0.33	0.33	0	0	0	8.5	0.2	11.8	0.2	15.0	0.2	7.3	0.2	11.1	0.9	12.7	0.1
18	0.33	0.33	0	0	0	4.8	0.1	7.4	0.1	11.5	0.2	4.6	0.1	6.8	0.1	10.2	0.1
19	0.33	0	0.33	0	0	8.6	0.4	11.7	0.5	15.2	0.4	8.1	0.2	11.4	0.5	13.2	0.6
20	0.33	0	0.33	0	0	28.4	0.0	34.3	0.2	42.3	1.1	24.3	0.5	30.2	0.6	35.3	1.0
21	0.33	0	0	0	0	39.2	0.8	44.8	0.3	50.6	2.1	34.0	0.8	41.0	0.4	44.1	3.8
22	0	0.33	0.33	0	0	8.9	0.2	12.9	0.5	15.5	0.4	8.6	0.2	12.2	0.2	14.6	0.9
23	0	0.33	0.33	0	0	27.2	0.1	33.0	0.4	39.8	0.7	22.9	0.9	28.6	0.5	35.5	1.1
24	0	0.33	0	0	0	38.2	0.3	43.9	0.2	47.6	0.9	32.5	0.4	40.4	1.8	44.0	3.3
25	0	0	0.33	0	0	39.2	1.4	43.3	1.1	46.1	1.0	35.1	2.7	37.8	2.8	40.5	0.9
26	0.25	0.25	0.25	0	0	7.9	0.1	11.0	0.1	14.1	0.1	7.2	0.1	9.8	0.2	12.3	0.2
27	0.25	0.25	0.25	0	0	28.9	0.7	35.2	0.3	42.6	0.4	24.0	0.9	30.3	0.5	35.8	1.0
28	0.25	0.25	0	0	0	39.2	0.8	44.5	0.6	48.8	1.1	36.3	1.7	38.7	1.1	42.3	0.9
29	0.25	0	0.25	0	0	41.2	0.3	45.3	0.3	49.9	1.0	40.6	3.8	42.8	0.8	46.6	1.5
30	0	0.25	0.25	0	0	39.9	0.2	43.9	0.7	49.3	0.9	36.7	1.2	40.9	1.6	47.0	2.5
31	0.2	0.2	0.2	0	0	39.5	0.1	45.3	0.6	49.2	1.0	37.5	1.1	43.0	1.4	47.9	2.1
32	Ctec2/Htec2 7/3					59.1	0.6	65.1	0.5	71.6	0.6	49.5	0.8	58.8	1.4	66.9	4.5

Global differences of sugar yields (with 0% to 13.5% (#26) glucose yield differences and 0% to 7% (#20) xylose yield differences) for EA-CS(−) and EA-CS(+) were observed among 31 enzyme combinations summarized in tables 2 and 3. This demonstrated that both core cellulases and hemicellulases were inhibited by alkali-soluble lignin in hydrolysis. Among all enzyme combinations from #1 to #31, the enzyme combination #26 (CBH I, CBH II, EG I, EX) gave the highest glucan conversion of 93.9% (at 30 mg g^−1^), 78.0% (at 15 mg g^−1^) and 60.8% (at 7.5 mg g^−1^) (table 2). While the highest xylan conversion rates for the three protein loadings were observed in experiments #21 (CBH I, EX, βX) with 50.6% conversion rate at 30 mg g^−1^, #31 (CBH I, CBH II, EG I, EX, βX) with 45.3% conversion rate at 15 mg g^−1^, and #29 (CBH I, EG I, EX, βX) with 41.2% conversion rate at 7.5 mg g^−1^ (table 3). These results showed that the enzymes CBH I, CBH II, EG I and EX played significant roles in the glucan conversion, while the enzymes CBH I, EX and βX played important roles in xylan conversion in CS. Moreover, the enzymes CBH I and EX were crucial enzymes for both glucan and xylan conversion.

For glucose yields of EA-CS(−) and EA-CS(+), distinct differences were observed in experiments # 3, 7, 10, 13, 14, 16, 19, 20, 22, 23, 25, 26, 27, 29, 30 and 31 (table 2). Note that all these enzyme mixtures contained the enzyme EG I. In experiments # 4, 8, 28, 29 the glucan conversion differences were much smaller because of absence of EG I in these enzyme cocktails. In other words, lignin removal increased the glucan conversion when the enzyme EG I was added to the enzyme cocktail. Since both the pretreated substrates had cellulose III, EG I with its open catalytic domain structure is preferred compared to CBH I and CBH II which have closed catalytic domains [43]. For all the individual enzymes (experiment #1 to #5) supplied with 10% βG, although the glucose yields were very low, we still observed statistically significant sugar conversions. When we compared the sugar yields of EA-CS(−) and EA-CS(+), the largest sugar conversion difference (3.4–4.3%) was observed in experiment #3 which had EG I enzymes supplemented with βG (table 2). In binary enzyme combinations (#6 to #15) with 10% βG supplementation, experiments # 7, 10, 11, 13 and 14 gave the biggest glucan conversion differences. In these experiments, EG I was present. Interestingly, the glucose yields also varied in experiment #6, suggesting that the lignin impacted the hydrolysis when CBH I and CBH II were combined in the enzyme cocktail, and EG I was not added. Similar result was seen in ternary enzyme combinations in experiments #17 and #18. Ternary enzyme combination experiments # 19, 20, 22, 23, 25 and quaternary enzyme combination experiments # 26, 27, 29, 30 all contained EG I. In all these cases, we observed significant differences in glucan conversion between EA-CS(−) and EA-CS(+). However, there were no significant differences observed for glucan conversions in experiments #21 (CBH I, EX, βX) and #28 (CBH I, CBH II, EX, βX), where the enzyme EG I was not contained. These results clearly showed that the enzyme activity of one of the core cellulases, EG I, was mostly inhibited by ammonia-soluble lignin during glucan conversion. Besides, by comparing the glucose yield variations of all 31 enzyme combinations for hydrolyzing EA-CS(−) and EA-CS(+), and analysing their statistical significance (*T*-test in electronic supplementary material, table S3), we could clearly see the conclusions for the effects of alkali-soluble lignin on mono- and multi-component enzymes, where EG I was inhibited mostly by lignin when compared with other individual enzymes (#3). EG I*βX was inhibited mostly by lignin in all the binary enzyme combinations (#14). CBH II*EG I*βX was inhibited mostly by lignin among all the ternary enzyme combinations (#23). CBH I*CBH II*EG I*βX was inhibited mostly by lignin when compared with all other quaternary enzyme combinations. Besides, the intensity of lignin inhibition for all the quaternary enzyme combinations was: CBH I*CBH II*EG I*βX > CBH I*CBH II*EG I*EX > CBH II*EG I*EX*βX > CBH I*EG I*EX*βX > CBH I*CBH II*EX*βX (#27).

Compared to glucan conversions, the differences between xylan conversions observed for the two different biomass materials were even more significant in enzyme combination experiments # 14, 15, 20, 21, 23, 24, 25, 27, 28, 29, 30, 31 as shown in table 3. For enzyme combinations that gave higher xylan conversions (greater than 20% at 7.5 mg g^−1^; greater than 25% at 15 mg g^−1^; greater than 30% at 30 mg g^−1^), at least one of the enzymes EG I or EX was present in the cocktail. In other words, xylan conversions were not significantly different unless either EG I or EX was added to the cocktail. There was only a very small xylan conversion difference (0.9%) in experiment #22, even when EG I and EX were added. Perhaps the absence of βX allows xylobiose to accumulate in the hydrolysate and hence it reduces xylan conversions. As soon as we added βX to the cocktail, we observed significant xylan conversion difference (5.6%) in experiment #25. Besides, in enzyme combination experiments (#20, 23, 27, 29, 30) with both high glucan and xylan conversions, distinct sugar conversion differences were observed, and at least one of the two enzymes EG I and EX were present in these enzyme combinations. This demonstrated that significant lignin inhibition appeared only when EG I or EX were included in the cocktail.

The differences of glucose and xylose yields for EA-CS(−) and EA-CS(+) using commercial enzymes (CTec:HTec at 7 : 3) (tables 2 and 3) were observed the same trend as that using the six core enzyme combinations. However, the highest xylan conversion for purified enzyme cocktail at 30 mg g^−1^ enzyme mass loading for EA-CS(−) was found to be much lower when compared to commercial enzymes (CTec:HTec at 7 : 3) (table 3). These results suggested that the six core enzyme combinations were sufficient to produce higher glucan conversion; however, we could achieve only 50% xylan conversion. Additional accessory enzymes are necessary to further improve the xylan conversion. Hemicellulose conversion is greatly influenced by accessory enzymes (e.g. arabinofuranosidase, galactanase, pectinesterase, fucosidase, esterase, rhamnosidase), because the structure of hemicellulose is more complex than that of cellulose.

### 3.3. Optimal core enzyme mixture for hydrolysing extractive ammonia-pretreated corn stover

Different enzyme combinations and the corresponding conversion results are given in tables 2 and 3. The results were analysed using Minitab designed statistically based predictive model to identify enzyme combinations that gave the highest glucan as well as xylan conversions. We plotted a response surface diagram of glucan conversion for both EA-CS(−) and EA-CS(+) for three protein loadings (7.5, 15 and 30 mg g^−1^ with 10% βG supplementation). The optimum enzyme cocktail that gave the highest glucan conversion was predicted using response surface curves. Optimal enzyme combinations that gave the highest predicted glucan conversion for three protein mass loadings are given in table 4. Model terms, associated correlation coefficients and *p*-values (less than 0.05) are listed in table 5 and electronic supplementary material, table S2 (for glucan and xylan conversions, respectively). Only terms that have a significantly low *p*-value (less than 0.05) have been included to develop statistically valid and highly predictive models. The coefficients of the model also provide insight into ranking the importance of each enzyme and synergistic interactions among various enzymes on the hydrolysis yields. The model developed in this work provided very good predictions, with regression *R*^2^ > 98%, *R*^2^ (pred.) > 97%, *R*^2^ (adj.) > 98%. The predicted optimal enzyme ratios were validated by enzyme hydrolysis experiments, and the results are shown in electronic supplementary material, table S4, with standard deviations.

**Table 4.** Optimized enzymes for EA-CS(−) and EA-CS(+) at three different enzyme loadings (7.5, 15 and 30 mg g^−1^ of glucan), with βG loading at 10% supplementation. Note: *p*-values < 0.05, *R*^2^ > 0.97. Enzyme loading was based on mg g^−1^ glucan. Experiments were done in triplicate to check their reproducibility.

	EA-CS(−)			EA-CS(+)		
purified enzymes	7.5 mg g^−1^ (%)	15 mg g^−1^ (%)	30 mg g^−1^ (%)	7.5 mg g^−1^ (%)	15 mg g^−1^ (%)	30 mg g^−1^ (%)
CBH I	27.2	27.3	28.2	28.2	28.1	27.3
CBH II	22.2	20.9	18.2	22.2	19.5	17.3
EG I	34.3	32.2	29.2	33.5	31.1	27.0
EX	9.0	10.3	14.1	9.1	12.2	16.1
βX	7.2	9.4	10.2	7.1	9.1	12.3

**Table 5.** Statistical model regression coefficients for glucan conversion at three protein mass loadings for EA-CS(−) and EA-CS(+) pretreated biomass. Coef., correlation coefficient.

	EA-CS(−) 7.5 mg g^−1^		EA-CS(−) 15 mg g^−1^		EA-CS(−) 30 mg g^−1^		EA-CS(+) 7.5 mg g^−1^		EA-CS(+) 15 mg g^−1^		EA-CS(+) 30 mg g^−1^	
purified enzyme and their combinations	Coef.	*p*-value	Coef.	*p*-value	Coef.	*p*-value	Coeff.	*p*-value	Coef.	*p*-value	Coef.	*p*-value
CBH I	0.06	*	0.12	*	0.23	*	0.05	*	0.10	*	0.22	*
CBH II	0.07	*	0.10	*	0.15	*	0.06	*	0.08	*	0.12	*
EG I	0.17	*	0.21	*	0.26	*	0.13	*	0.16	*	0.22	*
EX	0.05	*	0.07	*	0.09	*	0.05	*	0.06	*	0.08	*
βX	0.03	*	0.04	*	0.06	*	0.03	*	0.04	*	0.06	*
CBH I + EG I	0.99	0.00	1.52	0.00	1.98	0.00	1.02	0.00	1.57	0.00	1.90	0.00
CBH I + EX	0.22	0.00	0.54	0.00	1.09	0.00	0.21	0.00	0.57	0.00	1.16	0.00
CBH II + EG I	0.73	0.00	1.08	0.00	1.55	0.00	0.69	0.00	1.05	0.00	1.61	0.00
CBH II + EX	0.30	0.00	0.50	0.00	0.84	0.00	0.25	0.00	0.46	0.00	0.86	0.00
EG I + EX	0.20	0.00	0.22	0.00	0.28	0.00	0.12	0.04	0.15	0.01	0.21	0.01
EG I + βX	0.22	0.00	0.25	0.00	0.29	0.00	0.14	0.01	0.16	0.00	0.17	0.04
CBH I + CBH II + EG I	6.95	0.00	7.61	0.00	6.04	0.00	7.04	0.00	7.17	0.00	5.19	0.00
CBH I + CBH II+EX	4.56	0.00	7.18	0.00	9.38	0.00	4.61	0.00	7.42	0.00	7.16	0.00
CBH I + EG I + EX	5.17	0.00	6.02	0.00	6.23	0.00	5.01	0.00	5.77	0.00	4.46	0.00
CBH I + EG I + βX	3.00	0.00	4.75	0.00	7.61	0.00	2.95	0.00	4.85	0.00	6.45	0.00
CBH II + EG I + EX	3.05	0.00	3.43	0.00	3.53	0.00	3.24	0.00	3.52	0.00	3.09	0.00
CBH II + EG I + βX	1.96	0.00	2.96	0.00	4.77	0.00	1.74	0.00	2.81	0.00	4.43	0.00
CBH I + CBH II + EG I + βX	31.92	0.00	42.77	0.00	35.14	0.00	30.49	0.00	36.92	0.00	26.84	0.00
CBH I + CBH II + EX + βX	17.56	0.00	35.60	0.00	58.12	0.00	18.19	0.00	37.58	0.00	65.94	0.00
CBH I + EG I + EX + βX	19.28	0.00	27.99	0.00	31.59	0.00	21.94	0.00	31.70	0.00	50.34	0.00
CBH II + EG I + EX + βX	13.03	0.00	18.27	0.00	19.41	0.00	13.47	0.00	19.94	0.00	26.90	0.00
Regression *R*^2^	98.68%		99.32%		99.06%		98.58%		99.28%		98.91%	
*R*^2^ (pred.)	97.89%		99.02%		98.70%		97.69%		98.90%		98.42%	
*R*^2^ (adj.)	98.31%		99.13%		98.79%		98.19%		99.08%		98.61%	

The glucan and xylan conversions varied as a function of enzyme combinations due to synergistic interactions between enzymes. The correlation coefficients also helped rank the relative importance of each enzyme and the synergistic interactions among various enzymes during the hydrolysis of EA-CS. An interesting difference at all three enzyme mass loadings was observed regarding all the quaternary terms listed in table 5. For the enzyme combination CBH I*CBH II*EG I*βX, the correlation coefficient of EA-CS(−) was higher than that of EA-CS(+). However, for CBH I*CBH II*EX*βX, CBH I*EG I*EX*βX and CBH II*EG I*EX*βX, the trend was completely opposite, where the correlation coefficient of EA-CS(−) was lower than that of EA-CS(+). These results indicated that higher coefficient of hemicellulase was required in biomass containing more lignin, as both of two core hemicellulases EX and βX were present in the enzyme combinations above.

**Figure 2.**
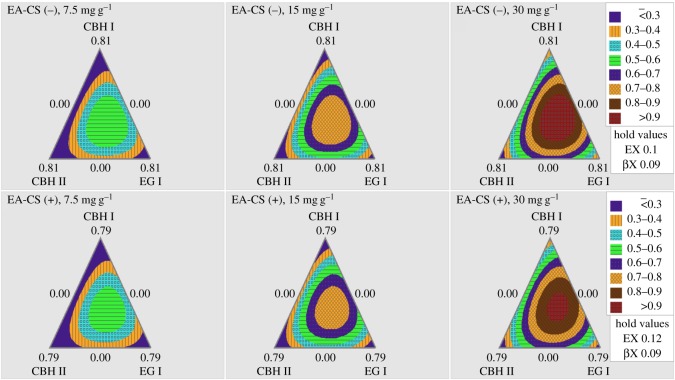
Ternary plot models of varying three enzymes (CBH I, CBH II, EG I) during hydrolysis that help to predict glucan conversion as a function of varying enzyme loadings. Two different pretreated CS (EA-CS(−) and EA-CS(+)) were used in these experiments. Expected glucan conversion ranges are denoted by the different colours/patterns as provided. Three different protein loadings (7.5, 15 and 30 mg g^−1^ of glucan) were used in these experiments.

**Figure 3.**
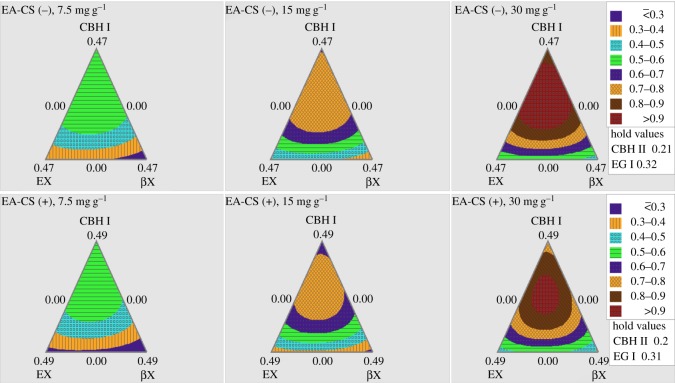
Ternary plot models of varying three enzymes (CBH I, EX, βX) during hydrolysis that help to predict glucan conversion as a function of varying enzyme loadings. Two different pretreated CS (EA-CS(−) and EA-CS(+)) were used in these experiments. Expected glucan conversion ranges are denoted by the different colours/patterns as provided. Three different protein loadings (7.5, 15 and 30 mg g^−1^ of glucan) were used in these experiments.

The contour plots for EA-CS(−) and EA-CS(+) are shown in figures 2 and 3, which give a pictorial representation of how varying enzyme ratios influences overall glucan conversion. The contour patterns of different protein loadings were quite congruous indicating that the optimum enzyme ratios were overlapping. When the enzymes EX and βX were fixed at their optimum ratios, all the three enzymes CBH I, CBH II and EG I were found to be important while varying enzyme ratios for glucan conversion. While fixing CBH I and CBH II absolute enzyme ratios, both EX and βX gave less significant functions among all three protein loadings.

**Figure 4.**
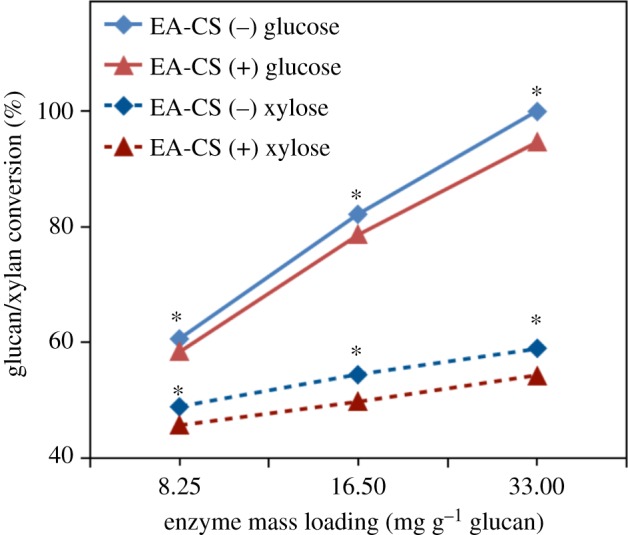
Sugar conversion when optimized enzyme loading was used for two different pretreated biomass, namely EA-CS(−) and EA-CS(+). Note: the statistical significance, *p ≤ 0.05.

The most remarkable difference observed in figure 2 was that CBH I, CBH II and EG I could achieve higher glucose yields on EA-CS(−) than those on EA-CS(+). Similar results were also observed for glucan conversion with the enzyme combination CBH I, EX and βX as shown in figure 3. These results showed that the glucan conversions using different enzyme combinations were affected by removing lignin from CS. In other words, we could achieve a higher glucan conversion at the same enzyme mass loadings for pretreated samples without lignin (figure 4).

**Figure 5.**
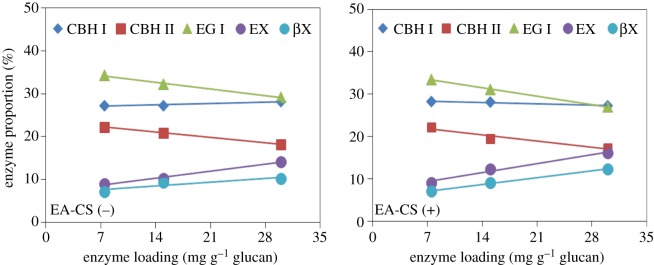
Changes in core enzyme requirement when we vary the enzyme loading. Here, percentages of core enzymes (CBH I, CBH II, EG I, EX and βX) that gave the highest glucan conversion at three enzyme loadings are given for two different pretreated CS, namely EA-CS(−) and EA-CS(+).

The optimal ratios of the six core enzymes that gave the highest glucan conversion varied among three different total enzyme loadings (8.25, 16.5, 33 mg protein g^−1^ glucan). The ranges of optimal enzyme ratios were: for EA-CS(−), CBH I: 27.2–28.2%, CBH II: 18.2–22.2%, EG I: 29.2–34.3%, EX: 9.0–14.1%, βX: 7.2–10.2%, βG: 1.0–5.0%; for EA-CS(+), CBH I: 27.3–28.2%, CBH II: 17.3–22.2%, EG I: 27.0–33.5%, EX: 9.1–16.1%, βX: 7.1–12.3%, βG: 0.5–5.0% (table 4). As the enzyme mass loading increased from 7.5 to 30 mg protein g^−1^ glucan, the same enzyme loading trend of core enzymes was observed for both EA-CS(−) and EA-CS(+) samples, but the optimal ratios of individual enzymes varied differently, the proportion of CBH I remained stable, the CBH II and EG I requirement in the cocktail decreased; however, the required level of EX and βX increased (figure 5).

Besides, the increase of EX and βX was greater in EA-CS(+) compared with EA-CS(−), showing that the hydrolysis of biomass without lignin extraction (EA-CS(+)) required higher proportions of EX and βX as the total enzyme loading increased. Perhaps when more enzymes are added to the substrate, most of glucan is converted into glucose, and much more oligomeric xylan is released in the solution phase, thus demanding more EX and βX in the cocktail to convert oligomeric sugars into monomeric sugars. Meanwhile, the enzymes CBH II and EG I decreased, because most of the glucan is converted into glucose. In addition, these two enzymes do not play a major role during the conversion of oligomeric xylan. The optimal CBH I proportion keeping stable might be because the enzyme CBH I still has minor effects on oligomeric xylan hydrolysis. Several studies have shown that oligomeric sugars are inhibitory to cellulases and hemicellulases [17,44,45].

**Figure 6.**
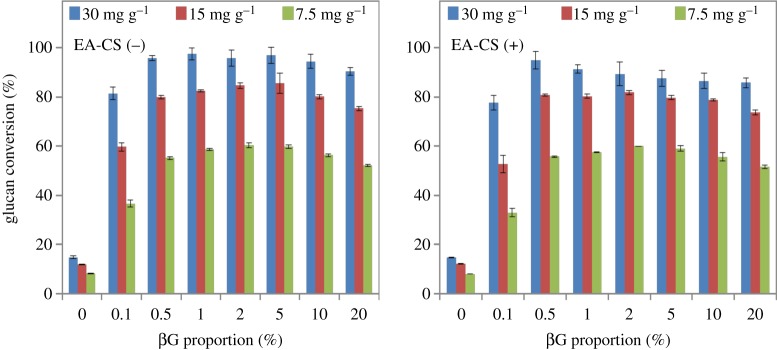
Varying concentration of βG with fixed amount of optimized core enzymes (CBH I, CBH II, EG I, EX and βX) at three total enzyme loadings for EA-CS(−) and EA-CS(+) pretreated biomass.

Results of the optimization of βG are shown in figure 6. In this experiment, we fixed the enzyme ratios of CBH I, CBH II, EGI, EX, βX at particular optimum mass loadings and varied the βG mass loading from 0% to 20% for both EA-CS(−) and EA-CS(+) samples. Results from these studies show that 0.5% to 1% of βG is sufficient to prevent inhibition of gluco-oligomers.

### 3.4. Lignin inhibition on optimal enzyme ratios

Activities of hydrolytic enzymes were affected differently by lignin from various sources and different pretreatment methods [46,47]. Although interactions between lignin and hydrolytic enzymes were investigated by previous studies [48,49], the effects of lignin form EA-CS on core enzymes is not reported in the literature. It is important to note that different thermochemical pretreatments produce different types of lignin which will have different effect on biomass-degrading enzymes during hydrolysis. In order to further understand the inhibitory role of alkali-soluble lignin on core cellulases and core hemicellulases at optimal enzyme ratios, we designed another set of experiments using pure Avicel and beechwood xylan and added water-insoluble, ethanol-soluble lignin fraction (WIL) isolated by EA extraction to the substrate before adding the enzyme cocktail [14]. Experiments were carried out at 1% Avicel and beechwood xylan mass loading, with lignin added at 1 : 5 (WIL : substrate weight). We also did another experiment by performing hydrolysis on EA-CS(−) without WIL addition and EA-CS(−) with WIL addition, while the biomass EA-CS(+) was used as control (here, with 0.4% glucan loading for EA-CS(−) and EA-CS(+), with WIL : biomass weight at 1 : 5). The enzyme cocktail used in this study was CBH I (25.9%), CBH II (19.8%), EG I (30.6%), EX (9.8%), βX (8.9%) and βG (5%) at 15 mg g^−1^ of glucan. These experiments show that both glucan and xylan conversions were affected by the presence of WIL isolated from EA extractives. These results are in agreement with previous studies where lignin extracted from liquid hot water pretreated sugar cane bagasse inhibited both cellulases and hemicellulases, particularly βG and βX were inhibited the most [50].

**Figure 7.**
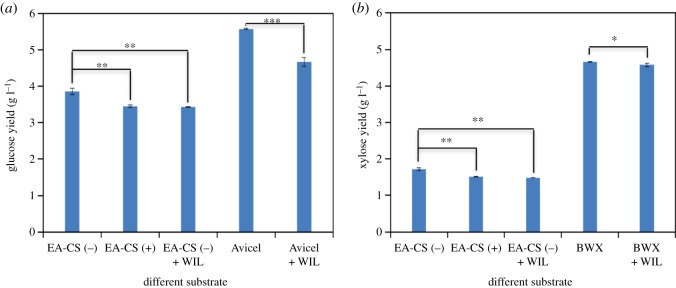
The effect of fractionated water-insoluble and ethanol-soluble lignin samples (WIL) on Avicel, beechwood xylan and EA-CS(−) sugar conversion is shown. (*a*) Glucan yield, (*b*) xylan yield. Hydrolysis experiment (24 h) was carried out using optimal enzymes (CBH I, 25.9%; CBH II, 19.8%; EG I, 30.6%; EX, 9.8%; βX, 8.9% and βG, 5%) at 15 mg g^−1^ of glucan. Experiments were carried out at 1% Avicel, beechwood xylan mass loading and 0.4% glucan loading for EA-CS(−) and EA-CS(+). Pure Avicel, beechwood xylan and EA-CS(−) were used as positive control and water insoluble lignin (WIL) was used as blank. The substrate to lignin ratio was kept at 5 : 1 in all the experiments, where lignin was added to the substrates before adding enzymes. The statistical significance: *p ≤ 0.05; **p ≤ 001; ***p ≤ 0.001.

The WIL inhibition of Avicel hydrolysis was much more pronounced than that for EA biomass (figure 7). This is probably due to the fact that the EA-CS sample contains cellulose III while Avicel contains highly crystalline cellulose I. Cellulose III is twice more digestible than cellulose I during enzyme hydrolysis [51]. In the case of xylan conversion, we noticed a small drop in conversion for the EA-CS(−) sample compared to EA-CS(+) samples. An even smaller drop in xylan conversion was noticed for beechwood xylan with the addition of WIL (figure 7). This could be due to the fact that WIL primarily acts on the solid reactant phase, and not on solubilized substrates. This hypothesis is in agreement with previous reported results which concluded that lignin isolated from lodgepole pine and steam pretreated poplar decreased the hydrolysis yields of Avicel, whereas the other isolated lignins did not appear to decrease the hydrolysis yields significantly [28]. Xylan when hydrolysed becomes soluble xylo-oligomers within the first few hours and most of the xylan-degrading enzymes (EX and βX) work in the solution phase to further deconstruct the xylo-oligomers to monomeric sugars.

## 4. Conclusion

Removal of alkali-soluble lignin and conversion of cellulose I to cellulose III both increase sugar conversions. We observed approximately 7–10% improvement in sugar conversion when alkali-soluble lignin was removed from CS. Both core cellulase and core hemicellulase are differently inhibited by ammonia-soluble lignin in biomass hydrolysis. The alkali-soluble lignin inhibits core cellulases more than it does core xylanases. The enzyme EG I is more inhibited by alkali-soluble lignin than other core cellulases. The lignin inhibition of the two core hemicellulases becomes more apparent at higher enzyme loadings using EA-treated biomass. The removal of ammonia-soluble lignin from CS could achieve more than 80% glucan conversion at 16.5 mg protein g^−1^ glucan enzyme dosage loading, while the conversion rate for xylan was around 50% at the same enzyme mass loading. This demonstrated that more accessory enzymes are necessary for efficient xylan conversion.

## Supplementary Material

Supplementary Tables

## Supplementary Material

Supplementary Figure S1

## Supplementary Material

Supplementary Figure S2

## Supplementary Material

Supplementary Figure S3

## Data accessibility

All necessary statistical data for this study are provided in the tables and supplementary tables, with these tables referred to in the text. And the data processing method is clearly described in §2.10.
