# Correction to: ‘The effect of alkali-soluble lignin on purified core cellulase and hemicellulase activities during hydrolysis of extractive ammonia-pretreated lignocellulosic biomass’

**DOI:** 10.1098/rsos.181213

**Published:** 2018-08-15

**Authors:** Linchao Zhou, Leonardo da Costa Sousa, Bruce E. Dale, Jia-Xun Feng, Venkatesh Balan

*R. Soc. open sci.* **5**, 171529. (Published 27 June 2018). (doi:10.1098/rsos.171529)

This correction refers to tables 2 and 3; the data of ‘EX’ and ‘βX’ (column; from nos 8 to 31) in these tables have been revised.

**Table 2.** Glucan conversion after 24 h hydrolysis for two different pretreated CS samples at three different protein loadings, while keeping βG loading at 10% supplementation in all experiments. Average (avg.) and standard deviation (s.d.) were based on experiments that were done in triplicate.

no.	purified enzymes					glucan conversion for EA-CS(−)						glucan conversion for EA-CS(+)					
						7.5 mg g^−1^		15 mg g^−1^		30 mg g^−1^		7.5 mg g^−1^		15 mg g^−1^		30 mg g^−1^	
	CBH I	CBH II	EG I	EX	βX	avg. (%)	s.d. (%)	avg. (%)	s.d. (%)	avg. (%)	s.d. (%)	avg. (%)	s.d. (%)	avg. (%)	s.d. (%)	avg. (%)	s.d. (%)
1	1	0	0	0	0	5.6	0.1	9.7	0.3	18.9	0.4	5.0	0.1	8.9	0.0	18.3	0.1
2	0	1	0	0	0	5.8	0.1	7.5	0.1	10.0	0.2	5.1	0.2	6.2	0.1	8.2	0.0
3	0	0	1	0	0	16.8	0.2	20.8	0.3	25.8	0.3	13.4	0.2	16.5	0.1	21.7	0.0
4	0	0	0	1	0	4.5	0.2	6.0	0.2	7.6	0.1	4.6	0.1	5.7	0.3	7.0	0.2
5	0	0	0	0	1	1.7	0.2	2.2	0.3	2.5	0.1	2.2	0.1	2.6	0.0	3.0	0.1
6	0.5	0.5	0	0	0	8.4	0.2	14.1	0.2	24.6	0.2	7.1	0.1	12.1	0.1	22.5	0.2
7	0.5	0	0.5	0	0	37.2	0.2	54.6	0.0	73.9	0.2	35.8	0.4	53.2	0.5	69.1	2.5
8	0.5	0	0	0.5	0	11.5	0.2	22.0	0.4	40.5	0.3	10.9	0.0	22.1	0.2	41.0	0.2
9	0.5	0	0	0	0.5	3.7	0.1	6.2	0.2	11.9	0.1	3.8	0.2	5.7	0.1	11.2	0.2
10	0	0.5	0.5	0	0	31.0	0.4	42.9	0.2	58.7	0.4	27.7	0.5	39.0	0.7	56.7	0.8
11	0	0.5	0	0.5	0	13.3	0.1	19.9	0.2	29.8	0.3	11.9	0.3	17.7	0.0	28.9	0.3
12	0	0.5	0	0	0.5	4.7	0.2	6.3	0.1	8.2	0.2	4.6	0.3	5.4	0.2	7.0	0.1
13	0	0	0.5	0.5	0	16.2	0.1	19.3	0.0	23.4	0.2	12.8	0.4	15.4	0.7	19.5	0.2
14	0	0	0.5	0	0.5	14.6	0.2	17.9	0.1	22.2	0.1	11.5	0.3	14.0	0.2	17.7	0.1
15	0	0	0	0.5	0.5	3.4	0.1	5.1	0.2	6.5	0.0	3.8	0.2	4.9	0.0	6.3	0.2
16	0.33	0.33	0.33	0	0	52.3	0.3	69.4	0.3	82.4	1.7	50.6	0.2	65.8	4.0	77.8	4.8
17	0.33	0.33	0	0.33	0	26.1	0.1	45.9	0.2	71.4	0.9	25.0	0.4	45.7	0.8	64.0	0.4
18	0.33	0.33	0	0	0.33	7.0	0.2	11.5	0.1	20.0	0.2	5.9	0.0	9.5	0.2	17.8	0.2
19	0.33	0	0.33	0.33	0	41.5	0.3	58.9	0.1	79.4	1.4	38.9	0.2	56.4	1.4	71.1	3.4
20	0.33	0	0.33	0	0.33	33.6	0.1	49.7	0.5	71.2	1.7	31.7	0.3	48.0	0.5	63.4	1.8
21	0.33	0	0	0.33	0.33	8.8	0.2	16.3	0.2	30.7	0.0	8.7	0.1	16.1	0.1	30.7	0.5
22	0	0.33	0.33	0.33	0	31.9	0.1	43.6	0.2	58.8	0.5	29.2	0.2	40.0	0.5	56.1	1.3
23	0	0.33	0.33	0	0.33	27.1	0.2	37.7	0.1	53.0	0.5	23.7	0.5	33.9	0.4	49.3	0.5
24	0	0.33	0	0.33	0.33	11.0	0.2	16.5	0.3	24.7	0.2	9.5	0.2	15.1	0.0	23.3	0.3
25	0	0	0.33	0.33	0.33	14.2	0.4	17.3	0.3	21.0	0.2	11.2	0.5	13.2	0.3	16.6	0.4
26	0.25	0.25	0.25	0.25	0	60.8	0.3	78.0	0.4	93.9	1.5	58.8	0.4	74.4	2.2	80.4	2.5
27	0.25	0.25	0.25	0	0.25	49.8	1.2	68.7	0.8	84.9	0.8	47.0	1.3	63.2	0.5	73.9	1.8
28	0.25	0.25	0	0.25	0.25	20.9	0.2	38.4	0.3	63.7	1.1	20.3	0.5	38.4	0.1	61.5	0.8
29	0.25	0	0.25	0.25	0.25	36.5	0.3	53.0	0.1	73.8	0.6	35.3	1.4	52.0	0.0	72.5	1.3
30	0	0.25	0.25	0.25	0.25	28.2	0.3	39.0	0.4	53.9	0.6	25.6	0.4	36.2	0.9	51.9	1.4
31	0.2	0.2	0.2	0.2	0.2	55.0	0.5	75.7	0.5	89.8	1.4	53.9	0.7	74.1	1.9	89.5	3.9
32	Ctec2/Htec2 7/3					73.5	0.1	88.5	0.7	99.5	1.4	65.5	1.3	81.0	0.7	94.1	1.9

**Table 3.** Xylan conversion after 24 h hydrolysis for two different pretreated CS samples at three different protein loadings, while keeping βG loading at 10% supplementation in all experiments. Average (avg.) and standard deviation (s.d.) were based on experiments that were done in triplicate.

no.	purified enzymes					xylan conversion for EA-CS(−)						xylan conversion for EA-CS(+)					
						7.5 mg g^−1^		15 mg g^−1^		30 mg g^−1^		7.5 mg g^−1^		15 mg g^−1^		30 mg g^−1^	
	CBH I	CBH II	EG I	EX	βX	avg. (%)	s.d. (%)	avg. (%)	s.d. (%)	avg. (%)	s.d. (%)	avg. (%)	s.d. (%)	avg. (%)	s.d. (%)	avg. (%)	s.d. (%)
1	1	0	0	0	0	2.3	0.1	3.6	0.1	5.8	0.3	2.1	0.0	3.3	0.0	5.3	0.1
2	0	1	0	0	0	1.3	0.1	1.5	0.2	2.1	0.2	1.5	0.2	1.7	0.1	2.2	0.2
3	0	0	1	0	0	6.9	0.5	10.7	0.2	14.2	0.4	6.6	0.2	9.8	0.1	13.1	0.1
4	0	0	0	1	0	13.4	0.7	19.3	0.7	24.2	0.6	12.5	0.4	17.2	1.1	22.5	1.5
5	0	0	0	0	1	2.4	0.2	2.8	0.4	3.3	0.1	2.4	0.1	2.9	0.2	3.2	0.1
6	0.5	0.5	0	0	0	1.8	0.2	2.9	0.1	4.3	0.3	2.0	0.1	2.9	0.1	4.4	0.2
7	0.5	0	0.5	0	0	5.5	0.1	8.3	0.2	11.0	0.3	5.1	0.2	7.6	0.3	10.3	0.2
8	0.5	0	0	0.5	0	10.6	0.4	14.6	0.2	18.1	0.5	8.9	0.2	13.3	0.2	16.2	0.4
9	0.5	0	0	0	0.5	5.5	0.2	8.3	0.1	12.3	0.1	5.1	0.2	7.3	0.1	11.1	0.2
10	0	0.5	0.5	0	0	5.6	0.2	8.3	0.1	11.3	0.2	5.2	0.2	7.2	0.3	10.5	0.3
11	0	0.5	0	0.5	0	10.7	0.3	15.0	0.1	18.3	0.2	9.7	0.5	12.8	0.6	16.8	0.7
12	0	0.5	0	0	0.5	2.3	0.1	3.3	0.2	4.1	0.1	2.6	0.2	3.3	0.5	4.0	0.1
13	0	0	0.5	0.5	0	11.1	0.1	15.4	0.2	19.3	0.4	9.8	0.8	14.1	1.6	19.1	0.2
14	0	0	0.5	0	0.5	29.3	0.2	33.7	0.3	39.5	0.3	24.7	0.6	29.7	0.5	34.7	0.6
15	0	0	0	0.5	0.5	40.3	0.7	45.0	0.9	48.6	0.7	36.6	3.4	40.9	0.5	45.0	2.6
16	0.33	0.33	0.33	0	0	4.8	0.1	7.2	0.1	10.1	0.1	4.3	0.1	6.3	0.2	9.3	0.1
17	0.33	0.33	0	0.33	0	8.5	0.2	11.8	0.2	15.0	0.2	7.3	0.2	11.1	0.9	12.7	0.1
18	0.33	0.33	0	0	0.33	4.8	0.1	7.4	0.1	11.5	0.2	4.6	0.1	6.8	0.1	10.2	0.1
19	0.33	0	0.33	0.33	0	8.6	0.4	11.7	0.5	15.2	0.4	8.1	0.2	11.4	0.5	13.2	0.6
20	0.33	0	0.33	0	0.33	28.4	0.0	34.3	0.2	42.3	1.1	24.3	0.5	30.2	0.6	35.3	1.0
21	0.33	0	0	0.33	0.33	39.2	0.8	44.8	0.3	50.6	2.1	34.0	0.8	41.0	0.4	44.1	3.8
22	0	0.33	0.33	0.33	0	8.9	0.2	12.9	0.5	15.5	0.4	8.6	0.2	12.2	0.2	14.6	0.9
23	0	0.33	0.33	0	0.33	27.2	0.1	33.0	0.4	39.8	0.7	22.9	0.9	28.6	0.5	35.5	1.1
24	0	0.33	0	0.33	0.33	38.2	0.3	43.9	0.2	47.6	0.9	32.5	0.4	40.4	1.8	44.0	3.3
25	0	0	0.33	0.33	0.33	39.2	1.4	43.3	1.1	46.1	1.0	35.1	2.7	37.8	2.8	40.5	0.9
26	0.25	0.25	0.25	0.25	0	7.9	0.1	11.0	0.1	14.1	0.1	7.2	0.1	9.8	0.2	12.3	0.2
27	0.25	0.25	0.25	0	0.25	28.9	0.7	35.2	0.3	42.6	0.4	24.0	0.9	30.3	0.5	35.8	1.0
28	0.25	0.25	0	0.25	0.25	39.2	0.8	44.5	0.6	48.8	1.1	36.3	1.7	38.7	1.1	42.3	0.9
29	0.25	0	0.25	0.25	0.25	41.2	0.3	45.3	0.3	49.9	1.0	40.6	3.8	42.8	0.8	46.6	1.5
30	0	0.25	0.25	0.25	0.25	39.9	0.2	43.9	0.7	49.3	0.9	36.7	1.2	40.9	1.6	47.0	2.5
31	0.2	0.2	0.2	0.2	0.2	39.5	0.1	45.3	0.6	49.2	1.0	37.5	1.1	43.0	1.4	47.9	2.1
32	Ctec2/Htec2 7/3					59.1	0.6	65.1	0.5	71.6	0.6	49.5	0.8	58.8	1.4	66.9	4.5

