# Supplementary material for: The effect of alkali-soluble lignin on purified core cellulase and hemicellulase activities during hydrolysis of extractive ammonia-pretreated lignocellulosic biomass
Source: R Soc Open Sci. 2018 Jun 27;5(6):171529. doi: 10.1098/rsos.171529 (PMC6030313; doi:10.1098/rsos.171529)
Supplement: Supplementary Tables [file rsos171529supp1.docx]

**The Effect of Alkali-soluble Lignin on Purified Core Cellulases and Hemicellulases Activities during Hydrolysis of Pretreated Lignocellulosic Biomass**

**Linchao Zhou^1^, Leonardo da Costa Sousa^2^, Bruce E. Dale^2^ Jia-Xun Feng^1*^ and Venkatesh Balan^1, 3*^.**

^1^ State Key Laboratory for Conservation and Utilization of Subtropical Agro-bioresources, College of Life Science and Technology, Guangxi University, Nanning, 530004, China

^2^ DOE Great Lakes Bioenergy Research Center(GLBRC), Biomass Conversion Research Laboratory(BCRL), Department of Chemical Engineering and Materials Science, Michigan State University, Lansing, MI 48910, USA

^3^Department of Engineering Technology, Biotechnology Division, School of Technology, University of Houston, Houston, TX 77004, USA.

* Correspondence: [vbalan@uh.edu](mailto:vbalan@uh.edu) and [jiaxunfeng@sohu.com](mailto:jiaxunfeng@sohu.com)

**Supplementary Tables**

**Table S1.** Core enzymes activities on different substrates.


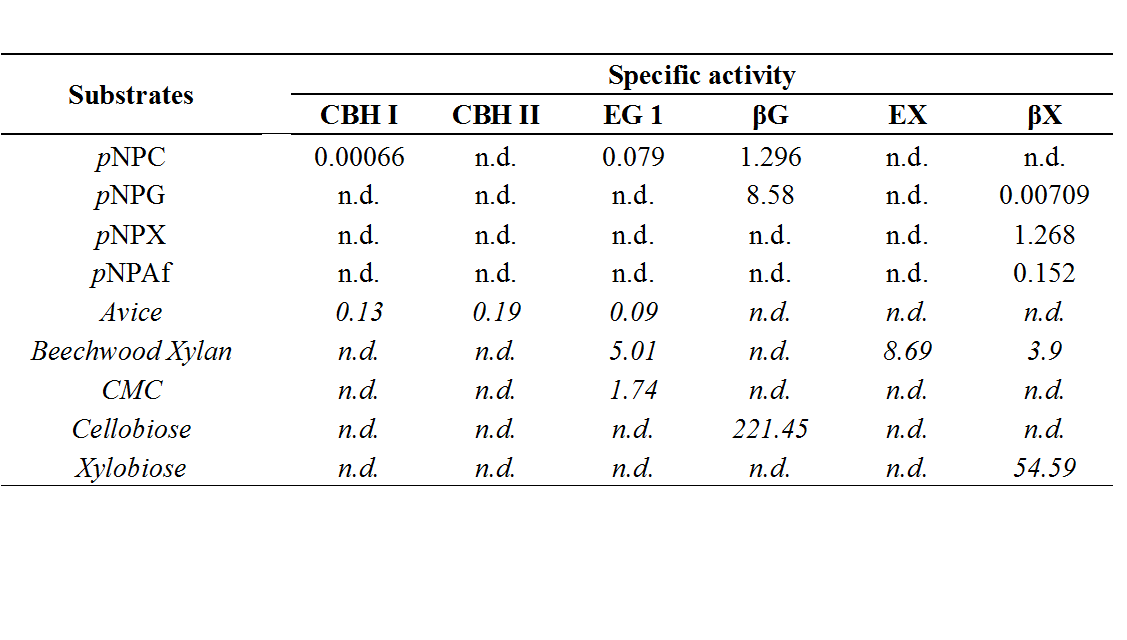


**Note:** One unit of specific activity was defined as 1 μmol *pNP* released per mg protein per minute. One unit of specific activity was defined as 1 μmol (as glucose equivalents) reducing sugars released based on DNS method (for Avicel and CMC) per mg protein per min. For beechwood xylan, specific activity was defined based on xylose equivalents. For cellobiose and xylobiose, one unit of specific activity was denoted as 1 μmol of glucose or xylose released per mg protein per minute, n.d. is not detectable.

**Table S2**. Statistical model regression coefficients for xylan conversion at three protein mass loadings for EA-CS(-) and EA-CS(+) pretreated biomass.


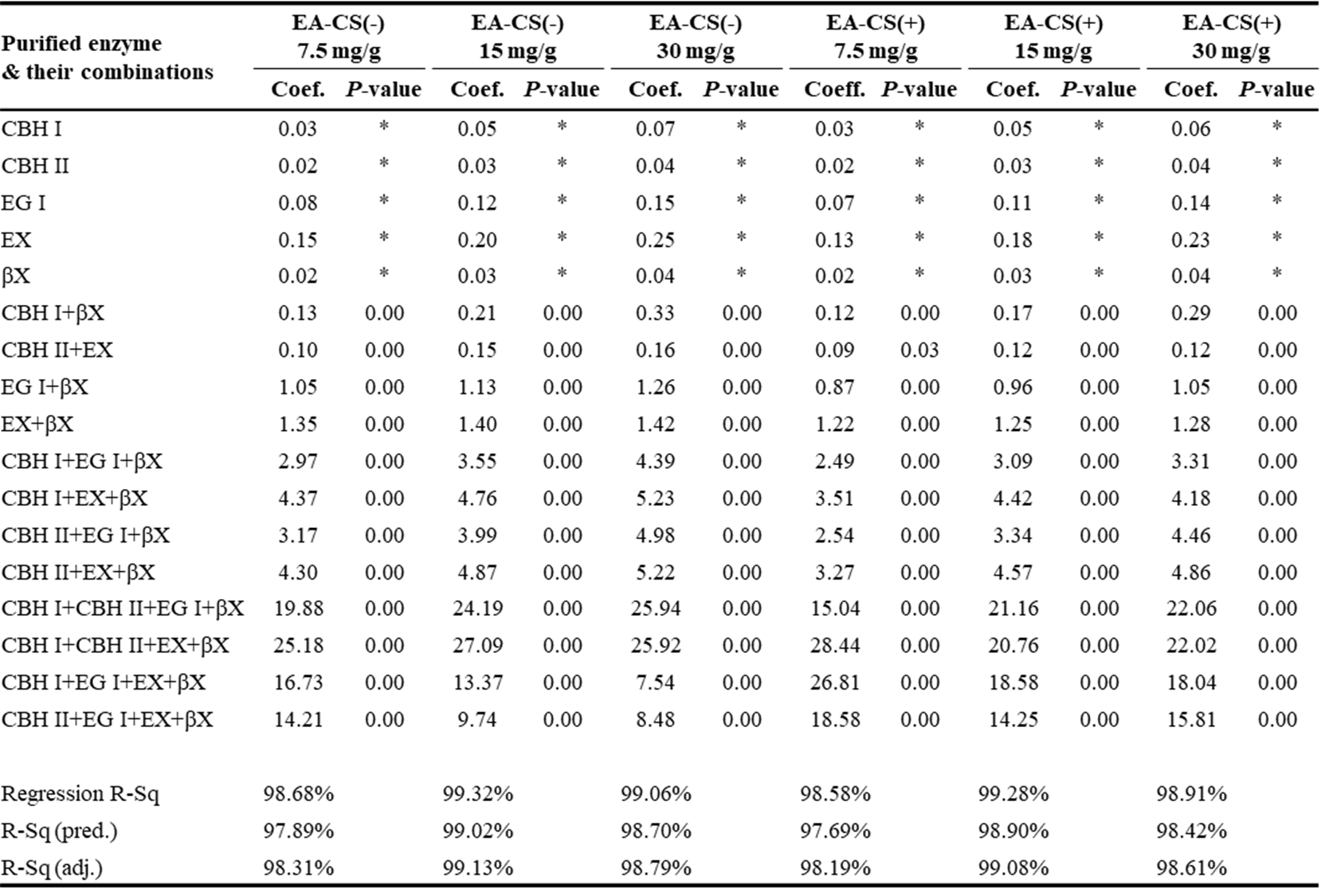


Note: Here, the Coef. is the correlation coefficient.

**Table S3.** The average difference of glucan conversion and their statistical significance for EA-CS(-) and EA-CS(+) under thirty-one enzyme combinations(at three different enzyme mass loadings, with 7.5, 15, and 30 mg protein/g glucan). Note: sig. here means significance. *: P<0.05; **: P<0.01; ***: P<0.001.


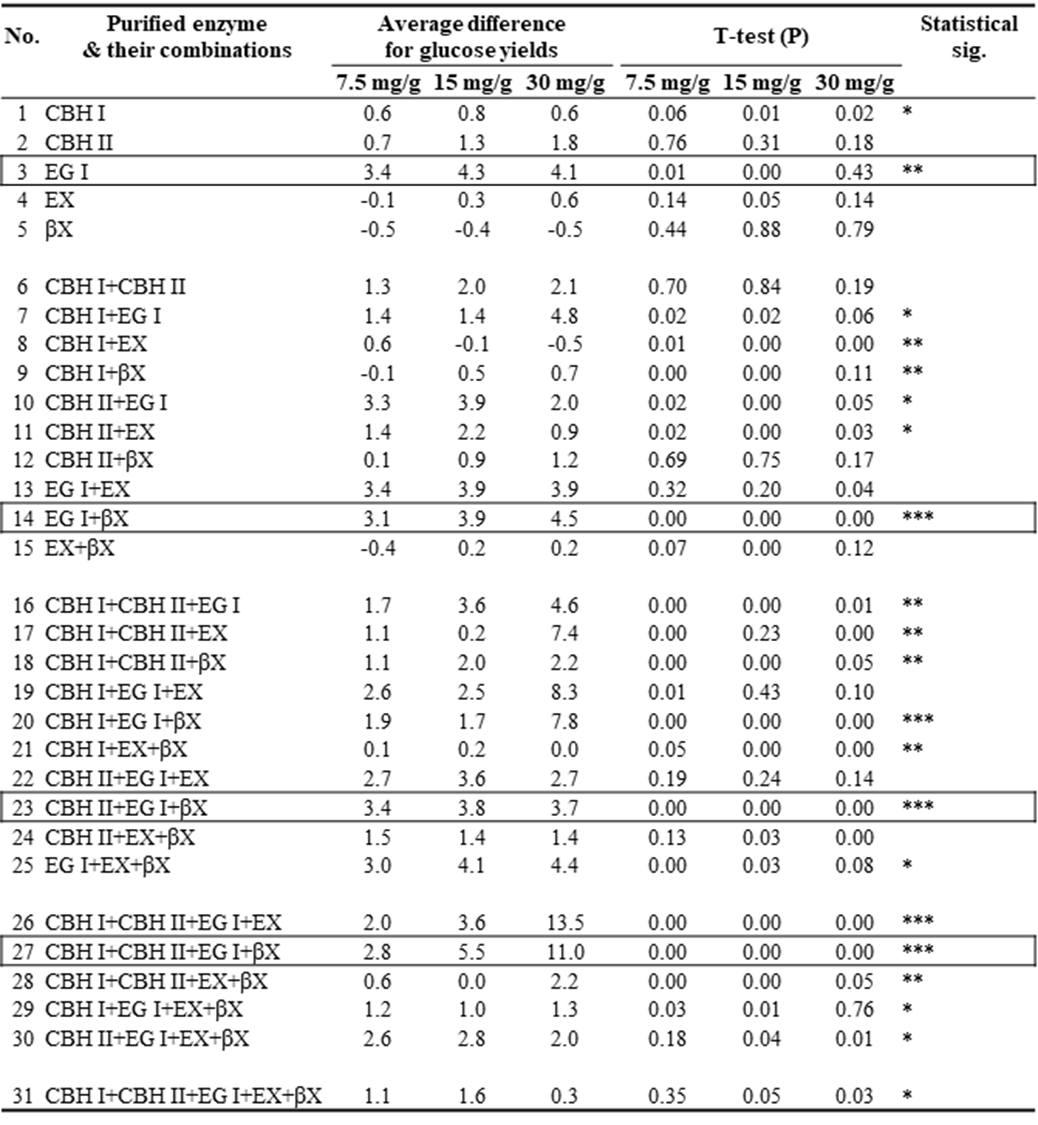


Note: 1. EG I was inhibited mostly by lignin when compared with other individual enzymes (#3).

2. EG I*βX was inhibited mostly by lignin in all the binary enzyme combinations (#14).

3. CBH II*EG I*βX was inhibited mostly by lignin among all the ternary enzyme combinations (#23).

4. CBH I*CBH II*EG I*βX was inhibited mostly by lignin when compared with all other quaternary enzyme combinations. Besides, the intensity of lignin inhibition for all the quaternary enzyme combinations are: CBH I*CBH II*EG I*βX > CBH I*CBH II*EG I*EX > CBH II*EG I*EX*βX > CBH I*EG I*EX*βX > CBH I*CBH II*EX*βX(#27).

**Table S4.** The model generated optimum mixture hydrolysis predictions were verified at three total enzyme loadings for both EA-CS(-) and EA-CS(+).


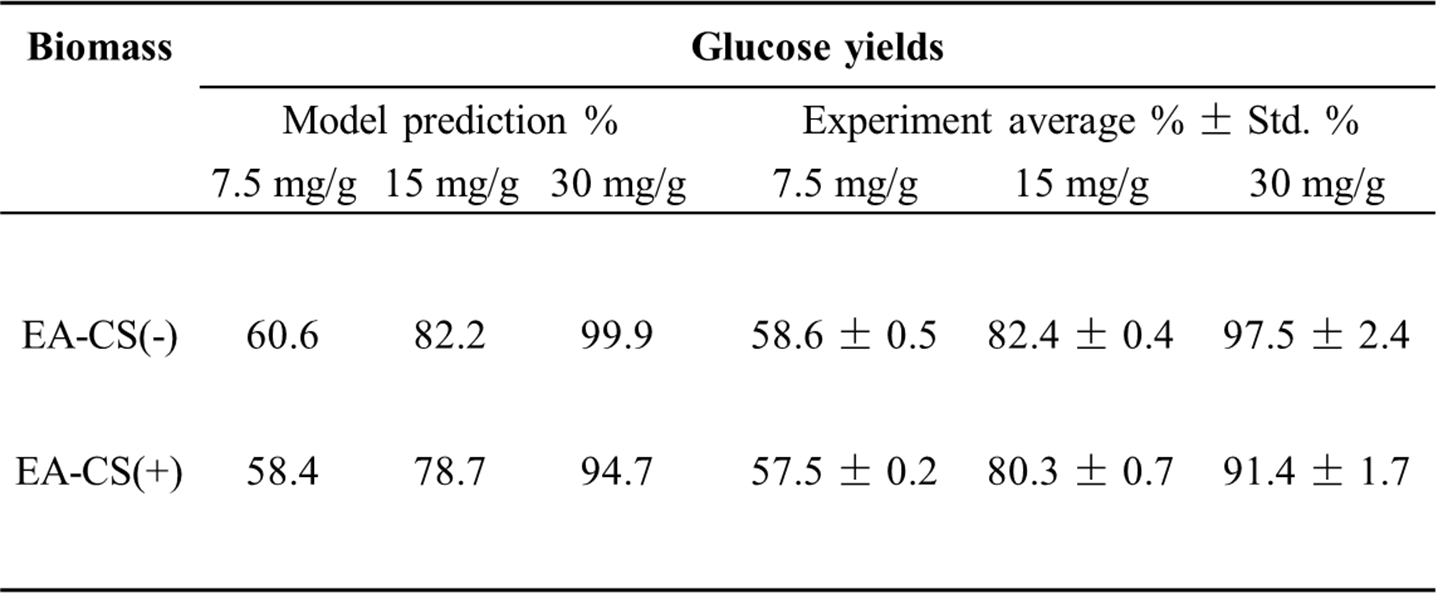


**Supplementary Figures**

**Fig. S1.** SDS-PAGE of purified biomass degrading enzymes used in all of the experiments. Here, EG I (lane 1), CBH II (lane 2), CBH I (lane 3), EX (lane 4), βX (lane 5) and βG (lane 6) and marker (lane M)

**Fig. S2.** Glucan conversion optimization using five core enzymes at different enzyme mass loadings (7.5 mg/g; 15 mg/g and 30 mg/g of glucan), with βG loading at 10% supplementation.

**Fig. S3.** Xylan conversion optimization using five core enzymes at different enzyme mass loadings (7.5 mg/g; 15 mg/g and 30 mg/g of glucan), with βG loading at 10% supplementation.
