# Supplementary figures and images for: The effect of alkali-soluble lignin on purified core cellulase and hemicellulase activities during hydrolysis of extractive ammonia-pretreated lignocellulosic biomass
Source: R Soc Open Sci. 2018 Jun 27;5(6):171529. doi: 10.1098/rsos.171529 (PMC6030313; doi:10.1098/rsos.171529)

kDa

M

1

2

3

4

5

6

190

120

85

60

50

40

25

20

15

10

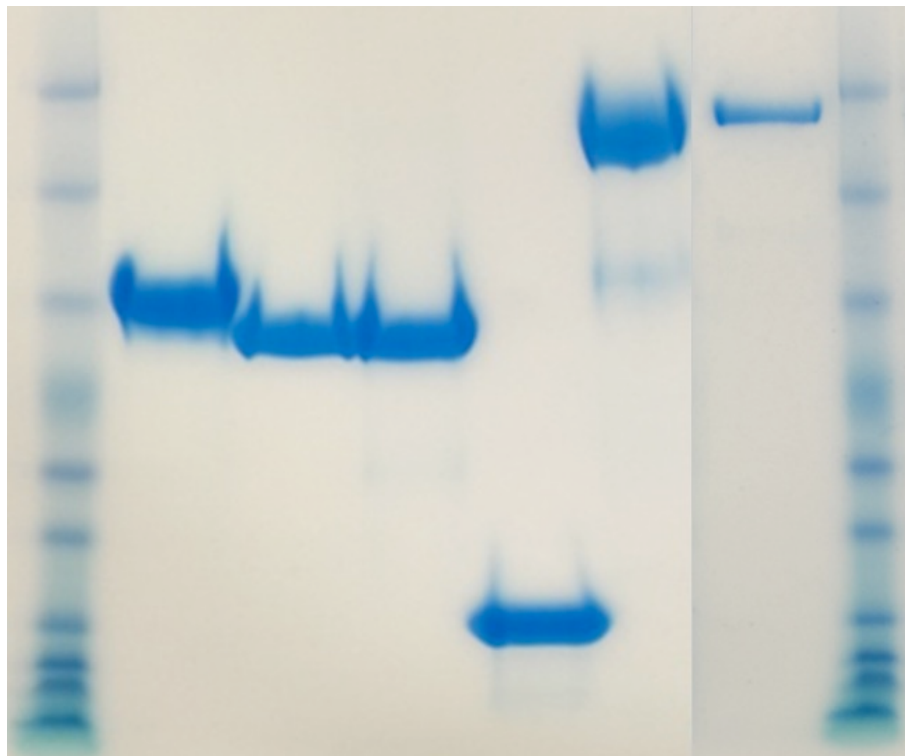

Supplement: Supplementary Figure S1 [file rsos171529supp2.pdf]

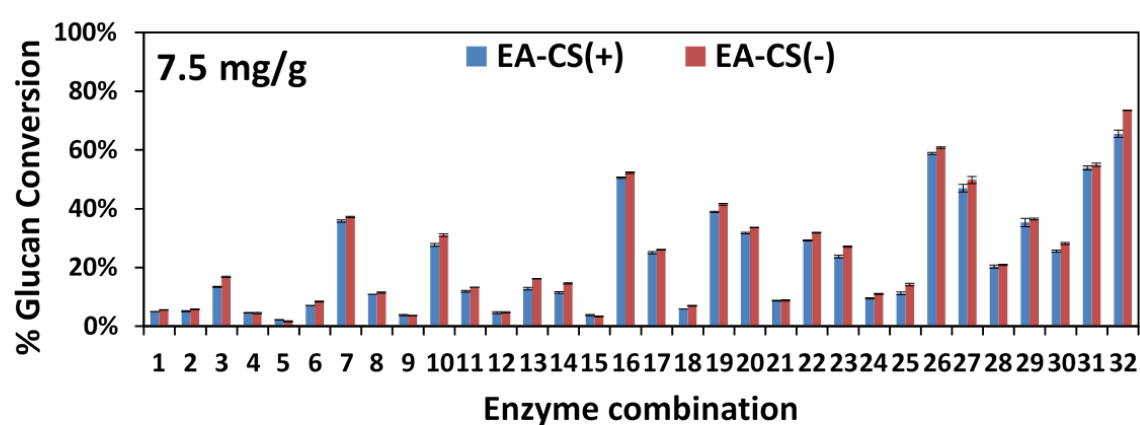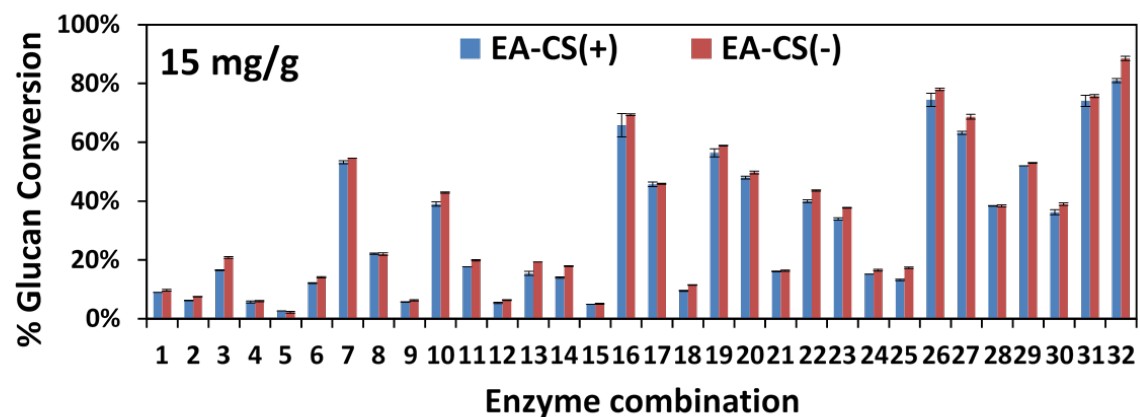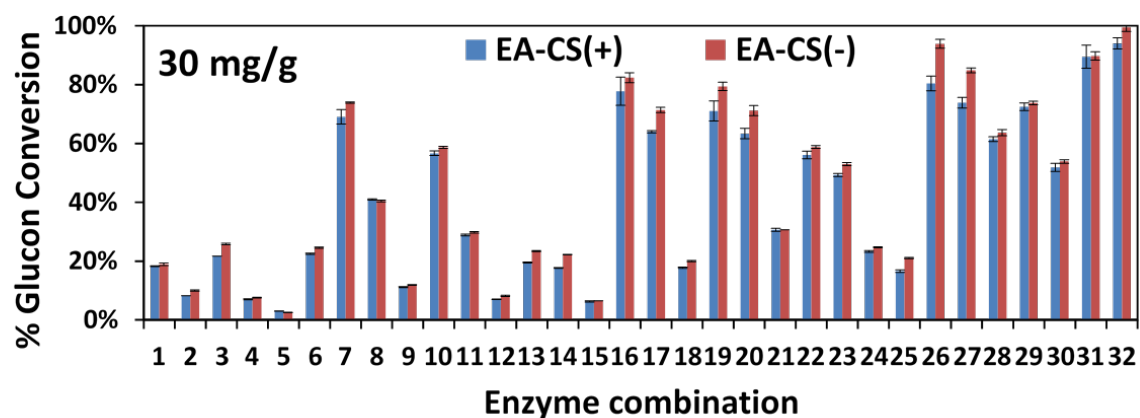

Supplement: Supplementary Figure S2 [file rsos171529supp3.pdf]

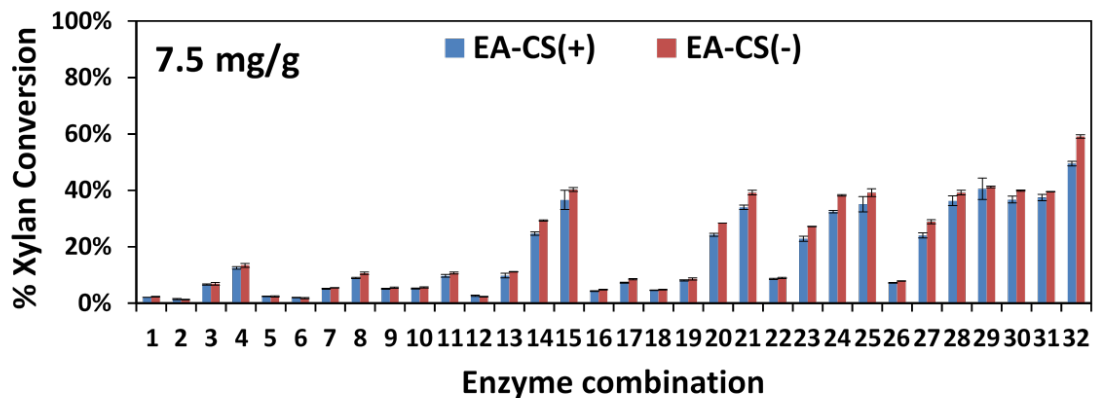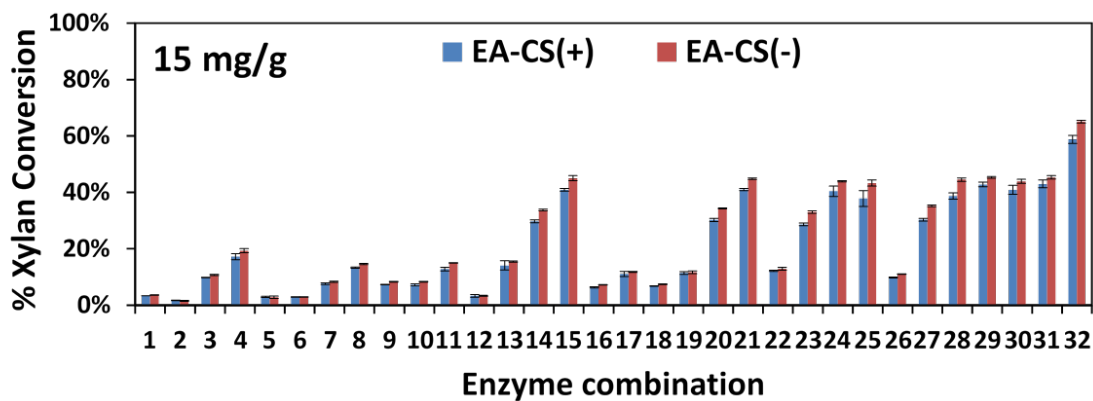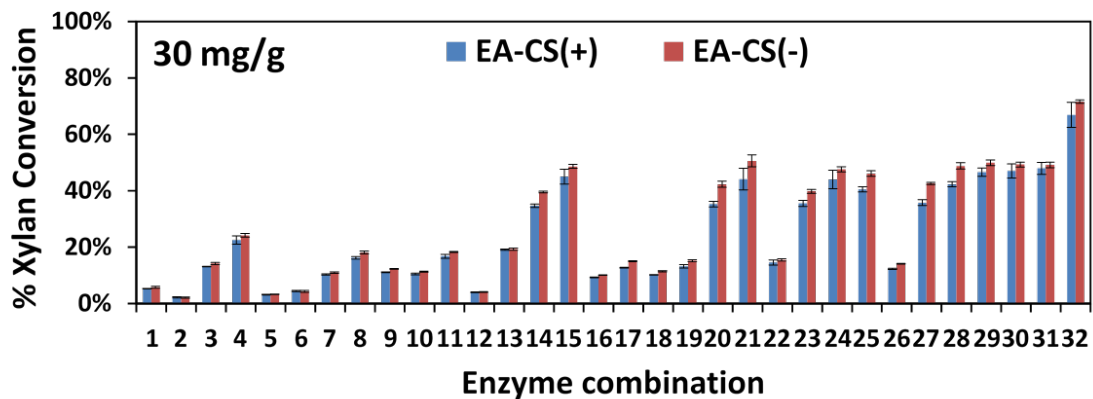

Supplement: Supplementary Figure S3 [file rsos171529supp4.pdf]
